# Spermidine restricts neonatal inflammation via metabolic shaping of polymorphonuclear myeloid-derived suppressor cells

**DOI:** 10.1172/JCI183559

**Published:** 2025-04-01

**Authors:** Jiale Chen, Lin Zhu, Zhaohai Cui, Yuxin Zhang, Ran Jia, Dongmei Zhou, Bo Hu, Wei Zhong, Jin Xu, Lijuan Zhang, Pan Zhou, Wenyi Mi, Haitao Wang, Zhi Yao, Ying Yu, Qiang Liu, Jie Zhou

**Affiliations:** 1 Tianjin Institute of Immunology, Key Laboratory of Immune Microenvironment and Disease (Ministry of Education), The Province and Ministry Co-sponsored Collaborative Innovation Center for Medical Epigenetics, International Joint Laboratory of Ocular Diseases (Ministry of Education), State Key Laboratory of Experimental Hematology, Department of Immunology, School of Basic Medical Sciences, Tianjin Medical University, Tianjin, China.; 2 Laboratory of Immunity, Inflammation and Cancer, Department of Oncology, The First Affiliated Hospital of Chongqing Medical University, Chongqing, China.; 3 Department of Clinical Laboratory, Children’s Hospital of Fudan University, National Children’s Medical Center, Shanghai, China.; 4 Department of Neonatal Surgery, Tianjin Children’s Hospital, Tianjin, China.; 5 Department of Neonatal Surgery, Guangzhou Women and Children’s Medical Center, Guangzhou Medical University, Guangzhou, China.; 6 Department of Oncology, The Second Hospital of Tianjin Medical University, Tianjin Key Laboratory of Precision Medicine for Sex Hormones and Diseases, Tianjin, China.; 7 Department of Pharmacology, Tianjin Key Laboratory of Inflammatory Biology, School of Basic Medical Sciences, Tianjin Medical University, Tianjin, China.; 8 Department of Neurology, Tianjin Neurological Institute, Tianjin Medical University General Hospital, Tianjin, China.

**Keywords:** Immunology, Metabolism, Innate immunity, Polyamines

## Abstract

Newborns exhibit a heightened vulnerability to inflammatory disorders due to their underdeveloped immune system, yet the underlying mechanisms remain poorly understood. Here we report that plasma spermidine is correlated with the maturity of human newborns and reduced risk of inflammation. Administration of spermidine led to the remission of neonatal inflammation in mice. Mechanistic studies revealed that spermidine enhanced the generation of polymorphonuclear myeloid-derived suppressor cells (PMN-MDSCs) via downstream eIF5A hypusination. Genetic deficiency or pharmacological inhibition of deoxyhypusine synthase (DHPS), a key enzyme of hypusinated eIF5A (eIF5A^Hyp^), diminished the immunosuppressive activity of PMN-MDSCs, leading to aggravated neonatal inflammation. The eIF5A^Hyp^ pathway was found to enhance the immunosuppressive function via histone acetylation–mediated epigenetic transcription of immunosuppressive signatures in PMN-MDSCs. These findings demonstrate the spermidine-eIF5A^Hyp^ metabolic axis as a master switch to restrict neonatal inflammation.

## Abstract 2

Spermidine restrains neonatal inflammation via eIF5A hypusination-mediated functional reprogramming of PMN-MDSCs.

## Graphical abstract

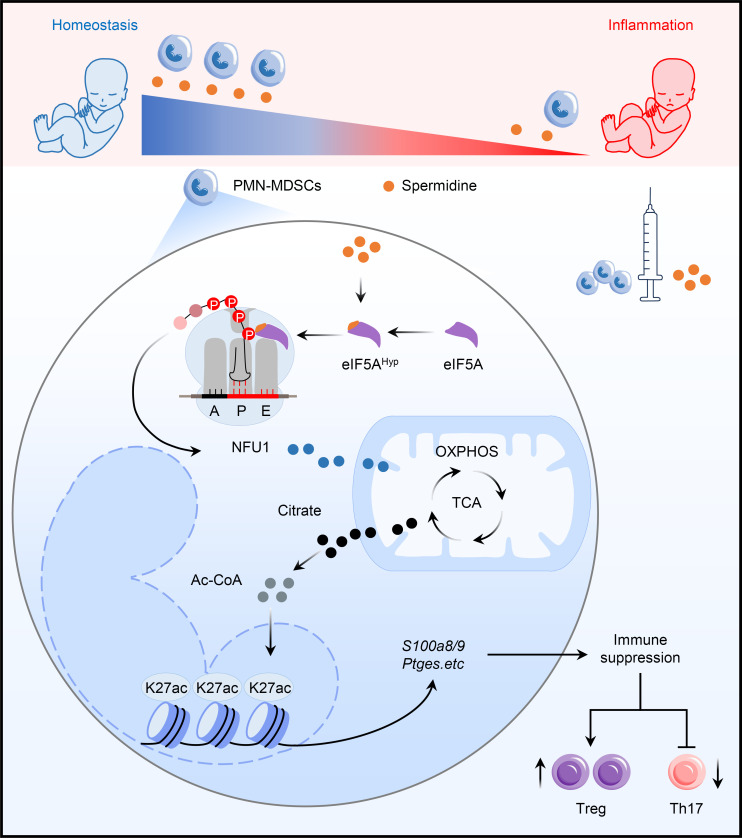

## Introduction

Neonatal mortality accounts for 47% of all deaths in children under the age of 5 years worldwide (1). A substantial portion of these deaths result from complications associated with preterm birth, particularly involving inflammatory diseases such as sepsis, necrotizing enterocolitis (NEC), and pneumonia (1, 2). The increased morbidity and mortality rates of inflammatory disorders in neonates are largely attributed to the immaturity of the postnatal immune system (3).

During early infancy, the maturation of the neonatal immune system is shaped by intrinsic developmental processes, as well as by external factors such as microbes, nutrients, and metabolites (4–6). Compared with adults, neonates exhibit greater immunotolerance, which leads to decreased efficiency of pathogen elimination and a reduction in the risk of excessive inflammation in response to microbiota colonization (3). Perturbation of neonatal immunity results in increased inflammation and tissue injury, such as NEC and sepsis, in infants (7–9). Although it has been suggested that feto-maternal tolerance or maternal-derived factors continue to influence the neonatal immune system after birth, the precise mechanisms underlying this immunotolerance in neonates have not been extensively studied (3, 10). The presence of specialized regulatory immune cells during the perinatal period is crucial for preserving immunotolerance in early life (11–14). We recently demonstrated that the transient appearance of regulatory myeloid cells, which are known as myeloid-derived suppressor cells (MDSCs), represents a mechanism contributing to this age-dependent immunotolerance in early infancy (7–9). Investigations into the fundamental mechanisms of neonatal immunotolerance may provide potential therapeutic approaches for neonatal inflammation.

After the cessation of nutrient supply from the cord blood at birth, infants must rely on the gastrointestinal system to obtain energy and nutrition in order to support the substantial requirements for growth, thermoregulation, and organ maturation during early infancy (4). This phenomenon has prompted inquiries into the manner in which the maturing immune system reacts to alterations in nutritional intake via metabolic adjustments, as well as the impact of cellular metabolism on the functionality of immune cells in neonates, and strategies for regulating metabolic pathways to promote immunotolerance and mitigate neonatal inflammation. The exploration of these inquiries may offer potential therapeutic approaches for managing neonatal inflammatory conditions.

Polyamines, including putrescine, spermidine, and spermine, play broad roles in health and in a variety of diseases (15). The levels of spermidine, which is the best-characterized polyamine, decrease with age, and its supplementation can improve or delay age-related pathologies (15). Spermidine has diverse protective effects on health, such as antiinflammatory and antioxidant effects, mitochondrial enhancement, and the induction of cytoprotective autophagy (15). Human breast milk and other mammal milks contain polyamines, which serve as crucial factors for luminal growth and maturation of the immune system (16, 17). However, the exact role of spermidine in neonatal health remains to be elucidated. In this study, compared with adults, newborns demonstrated a significant elevation in spermidine, which was correlated with a reduced risk of inflammation in human newborns. Spermidine was shown to enhance the immunosuppressive function of polymorphonuclear myeloid-derived suppressor cells (PMN-MDSCs) via eIF5A hypusination–mediated mitochondrial-epigenetic interactions. Supplementation with spermidine or spermidine-generated PMN-MDSCs demonstrated efficacy in the remission of neonatal inflammation. These observations reveal a previously unrecognized role of spermidine metabolism in the control of neonatal inflammation.

## Results

### Plasma spermidine is correlated with a reduced risk of neonatal inflammation in humans.

**Figure 1:**
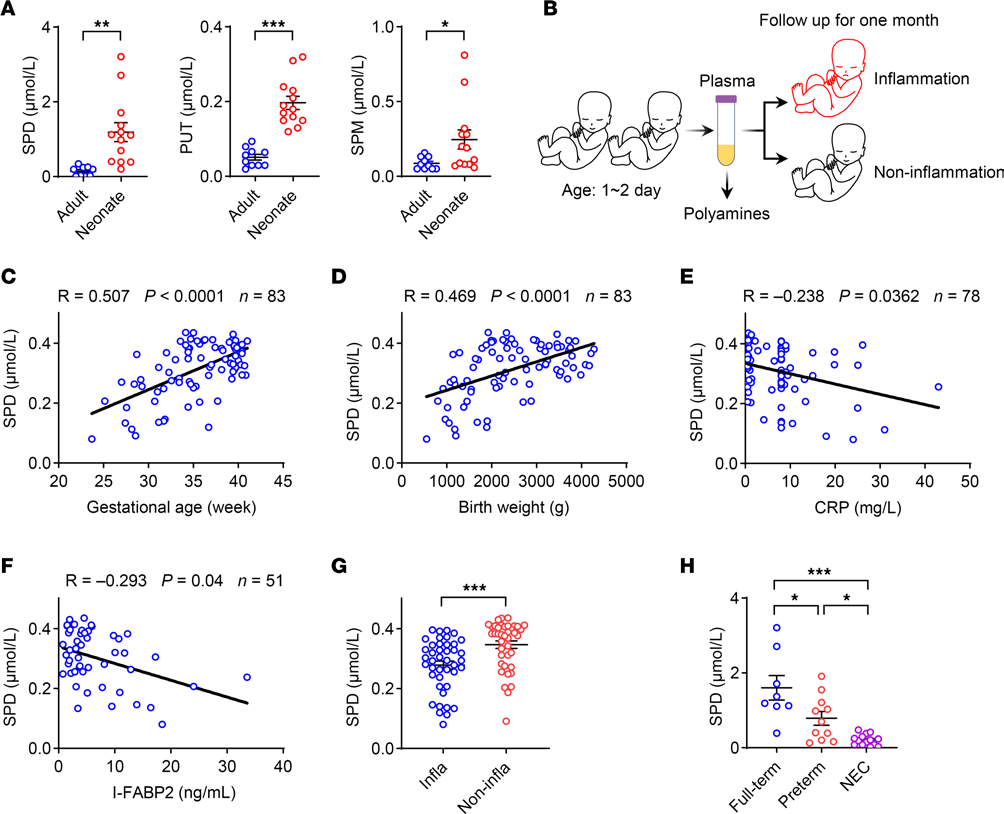
Plasma spermidine is correlated with a reduced risk of inflammation in human newborns. (**A**) Plasma polyamines in healthy adults (*n* = 10) and neonates (*n* = 13) were determined by UPLC-MS/MS. Clinical parameters are listed in Supplemental Table 1. SPD, spermidine; PUT, putrescine; SPM, spermine. (**B**–**G**) Plasma samples were collected from healthy neonates within 2 days of birth, and clinical parameters are listed in Supplemental Table 2. Polyamines and I-FABP2 were detected by ELISA. (**B**) Schematic approach for plasma sample collection. (**C**–**F**) Correlation between SPD and gestational age (**C**), birth weight (**D**), C-reactive protein (CRP) (**E**), and I-FABP2 (**F**). The infants were divided into 2 groups based on the presence of subsequent inflammatory disorders within 1 month of follow-up: the inflammation group and the non-inflammation group. (**G**) SPD concentration in inflammation group (Infla) (*n* = 42) and non-inflammation group (Non-infla) (*n* = 41). (**H**) Plasma SPD concentration in healthy full-term babies (*n* = 8), preterm babies (*n* = 11), and necrotizing enterocolitis (NEC) patients (*n* = 14). Clinical parameters are listed in Supplemental Table 1. Data are shown as mean ± SEM. Statistical analysis was performed using unpaired 2-tailed Student’s *t* test (**A** and **G**), Spearman’s correlation coefficient (**C**–**F**), and 1-way ANOVA with Tukey’s multiple-comparison test (**H**). *P < 0.05, **P < 0.01, ***P < 0.001.

To evaluate the potential role of polyamines in postnatal immunity, the concentrations of plasma polyamines in human infants and adult controls were determined by ultra-performance liquid chromatography–tandem mass spectrometry (UPLC-MS/MS). Results revealed that the levels of polyamines, including spermidine, putrescine, and spermine, were significantly elevated in infants compared with adults (Figure 1A and Supplemental Table 1; supplemental material available online with this article; https://doi.org/10.1172/JCI183559DS1). Similar observations were obtained in neonatal mice (Supplemental Figure 1A). We subsequently enrolled human newborns within 2 days of birth and followed them for 1 month to evaluate the clinical significance of polyamines in newborns (Figure 1B and Supplemental Table 2). Results showed that the levels of spermidine and spermine at birth were positively correlated with gestational age and birth weight (Figure 1, C and D, and Supplemental Figure 1, B and C), the clinical parameters reflecting infant maturity. Importantly, negative correlations were observed between plasma spermidine and the inflammation biomarker C-reactive protein, as well as the intestinal barrier function biomarker intestinal fatty acid–binding protein 2 (I-FABP2), at birth (Figure 1, E and F, and Supplemental Figure 1, D and E). These observations indicate that polyamine metabolism is associated with the maturation of human newborns at the early postnatal stage.

Infants are highly susceptible to inflammatory disorders, particularly those born prematurely or with low birth weights (18). Therefore, it is crucial to predict inflammation before its clinical manifestation. To assess the significance of plasma polyamines at birth in predicting future susceptibility to inflammation, the enrolled infants were divided into 2 groups based on the presence of subsequent inflammatory disorders (such as pneumonia, urinary tract infection, sepsis, NEC, etc.) within 1 month of follow-up: the inflammation group and the non-inflammation group. Results revealed that infants from the inflammation group exhibited lower levels of spermidine at birth than those from the non-inflammation group (Figure 1G and Supplemental Figure 1F), suggesting that spermidine levels at birth may reflect the susceptibility to neonatal inflammation. Furthermore, compared with age-matched full-term infants, NEC patients and preterm infants exhibited lower levels of spermidine (Figure 1H, Supplemental Figure 1G, and Supplemental Table 1). These findings indicate a potential role of polyamine metabolism in neonatal inflammation.

### Spermidine attenuates neonatal inflammation.

**Figure 2:**
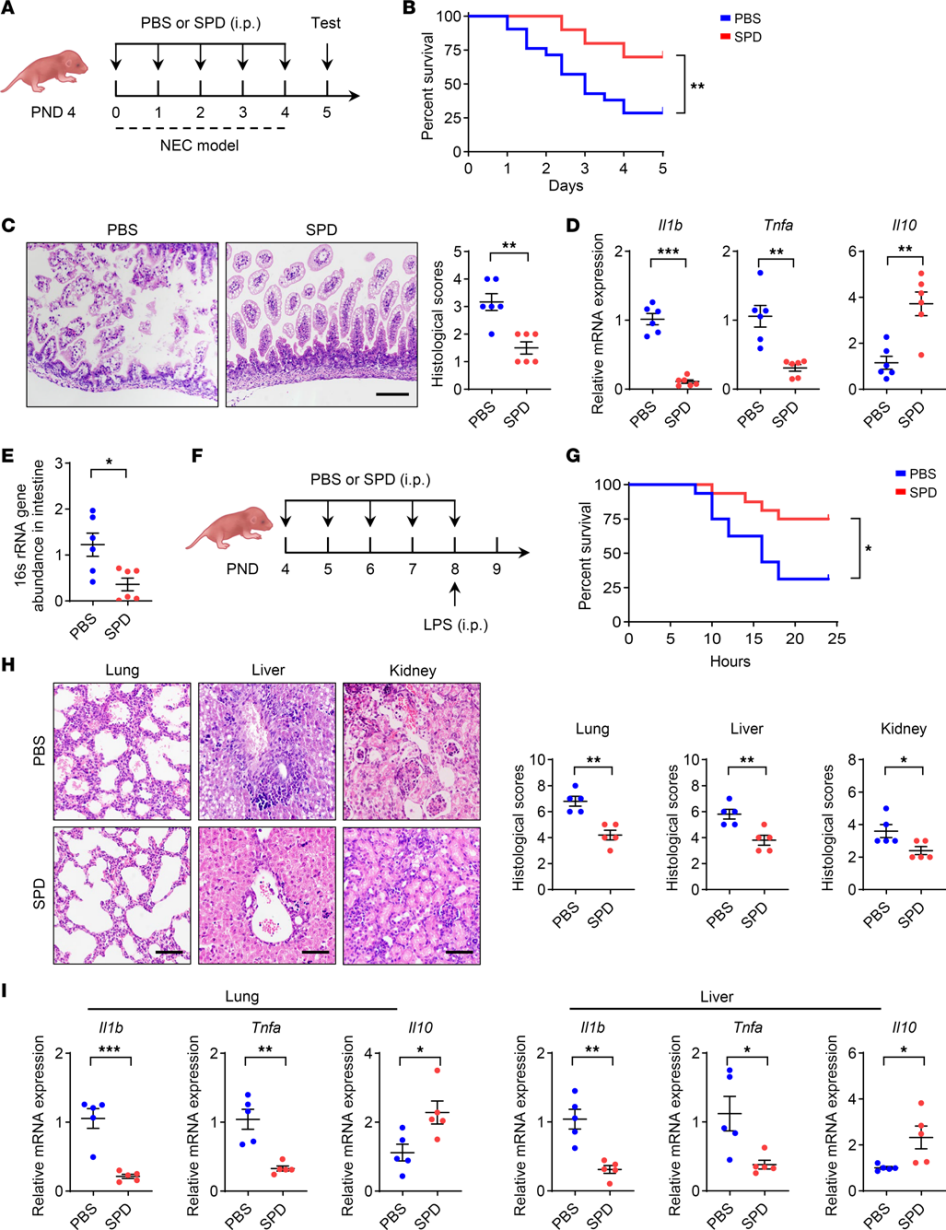
Spermidine attenuates neonatal inflammation. (**A**–**E**) Spermidine (SPD) for treating NEC. (**A**) Schematic approach for supplementation of SPD (10 mg/kg/d) during NEC induction. PND, postnatal day. (**B**) Survival curve of mice after induction of NEC (PBS, *n* = 21; SPD, *n* = 20). Data combined from 2 independent experiments. (**C**) H&E staining and histopathological scores of intestinal tissues (*n* = 6 per group). (**D**) Relative mRNA expression of *Il1b*, *Tnfa*, and *Il10* was determined by quantitative real-time PCR (*n* = 6 per group). (**E**) Bacterial abundance in the intestinal wall was evaluated by 16S abundance (*n* = 6 per group). (**F**–**I**) SPD for treating neonatal endotoxemia. (**F**) Schematic approach for supplementation of SPD (10 mg/kg/d); endotoxemia was induced by intraperitoneal injection of LPS (15 mg/kg). (**G**) Survival curve of mice after induction of endotoxemia (*n* = 16 per group). Data combined from 2 independent experiments. (**H**) H&E staining and histopathological scores of lung, liver, and kidney (*n* = 5 per group). (**I**) Relative mRNA expression of *Il1b*, *Tnfa*, and *Il10* was determined by quantitative real-time PCR (*n* = 5 per group). Data are shown as mean ± SEM. Statistical analysis was performed using log-rank (Mantel-Cox) test (**B** and **G**) and unpaired 2-tailed Student’s *t* test (**C**–**E**, **H**, and **I**). Scale bars: 100 μm. *P < 0.05, **P < 0.01, ***P < 0.001.

To examine the potential impact of spermidine on neonatal inflammation, spermidine was administered intraperitoneally to 4-day-old mice during the course of NEC induction (Figure 2A and Supplemental Figure 2A). Results showed that spermidine administration significantly ameliorated the severity of NEC, as represented by an improved survival rate (Figure 2B), reduced intestinal damage, and decreased histological scores (Figure 2C). Meanwhile, the expression of proinflammatory genes (*Il1b* and *Tnfa*) in intestines was downregulated and that of an antiinflammatory gene (*Il10*) was upregulated following spermidine treatment (Figure 2D). Additionally, there was a reduction in bacterial abundance within the intestinal wall (Figure 2E). Furthermore, spermidine-treated NEC pups exhibited a decreased proportion of type 17 helper T cells (Th17) and an increased proportion of regulatory T cells (Tregs) compared with the control group (Supplemental Figure 2B). Oral administration of spermidine via gavage also significantly reduced the severity of NEC (Supplemental Figure 2, C–G). We also evaluated the post-treatment effect of spermidine on NEC. After the completion of the NEC induction procedure, spermidine was administered to the affected mice. Results indicated that the post-treatment administration of spermidine exhibited comparable therapeutic effects on NEC (Supplemental Figure 2, H–L). In addition, spermidine did not influence the total amount of bacteria in intestines under steady-state conditions (Supplemental Figure 2M). These observations demonstrate that spermidine administration causes remission of NEC.

To determine whether the protective effect of spermidine is systematic, a lipopolysaccharide-induced (LPS-induced) neonatal endotoxemia model was established (Figure 2F and Supplemental Figure 2A). In line with the observations from the NEC model, the mortality of endotoxemic pups was significantly reduced following spermidine administration (Figure 2G). The mitigation of inflammation was further evidenced by diminished tissue damage (Figure 2H), as well as downregulation of proinflammatory genes (*Il1b* and *Tnfa*) and upregulation of the antiinflammatory gene *Il10* (Figure 2I) in multiple organs. This protective effect was further confirmed when spermidine was administered after endotoxemia was completed (Supplemental Figure 2, N–Q). These results collectively demonstrate that spermidine systematically mitigates neonatal inflammation.

### Polyamine metabolism is enriched in neonatal myeloid cells.

To explore the potential role of spermidine in neonatal immunity, we next evaluated the correlations between polyamines and the frequencies of immune cells in human newborns. Results revealed that the concentrations of plasma spermidine were positively correlated with the absolute cell counts of neutrophils, but not with other types of immune cells (Supplemental Figure 3, A–D). These observations suggest that polyamine metabolism may affect the biological function of neonatal neutrophils. We subsequently analyzed the single-cell RNA sequencing data of human neutrophils (19). A total of 5 clusters of neutrophils were identified. The immunosuppressive score was highest in cluster 2 (C2) (Supplemental Figure 3E), based on the MDSC signature genes. The COMPASS algorithm was used to examine the correlation between polyamine metabolism and neutrophil functionality; the Recon 2 metabolic models were used for metabolic activities (20). The genes encoding enzymes related to polyamine metabolism were annotated within the urea cycle pathway in this model. Results indicated a positive correlation between the activity of polyamine metabolism and the immunosuppressive score of neutrophils (Supplemental Figure 3F). These observations further indicate the potential role of polyamines in the functionality of immunosuppressive neutrophils in neonates.

**Figure 3:**
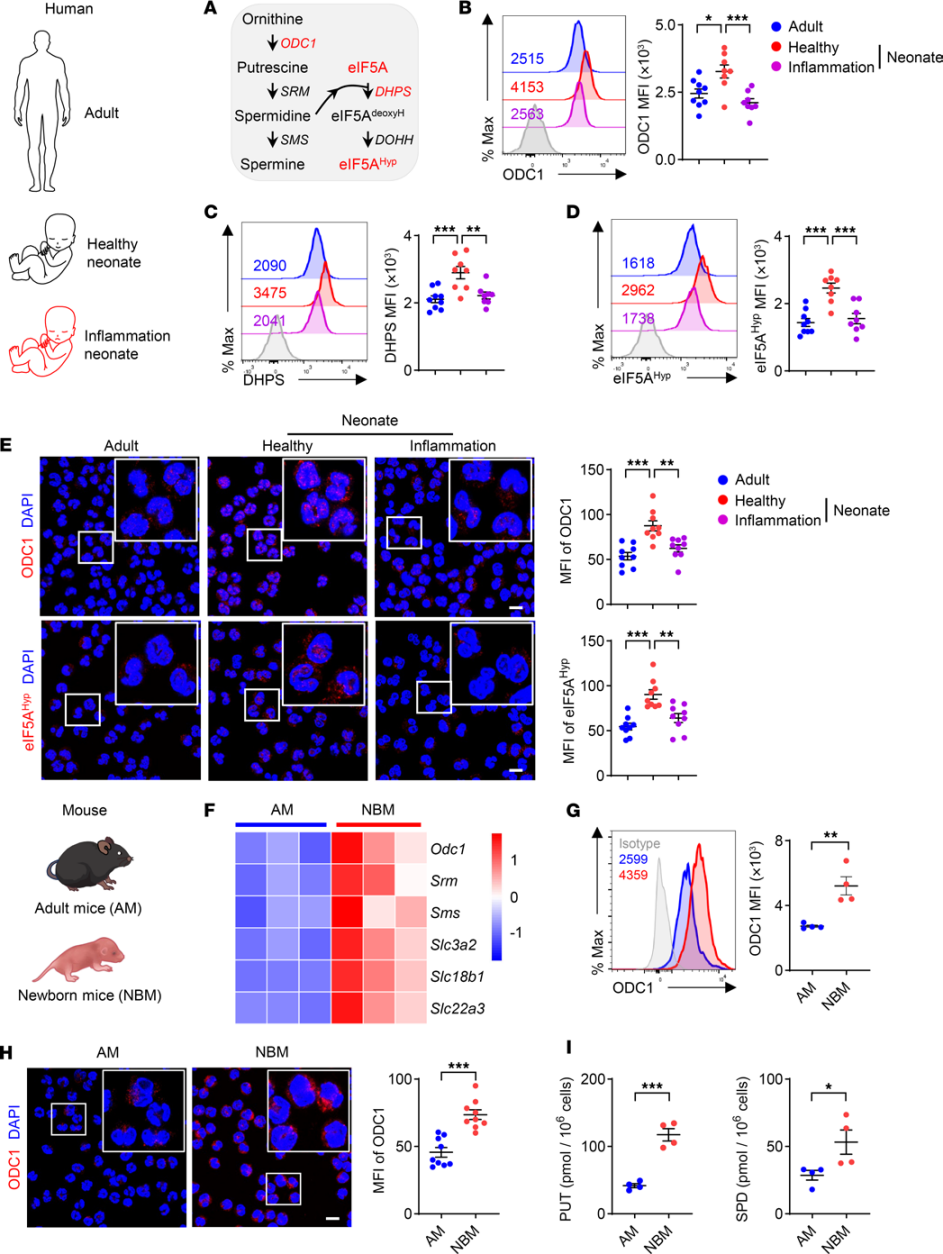
Polyamine metabolism is enriched in neonatal myeloid cells. (**A**) Polyamine metabolic pathway. (**B**–**D**) The expression of ODC1 (**B**), DHPS (**C**), and eIF5A^Hyp^ (**D**) in neutrophils (CD11b^+^CD14^–^CD15^+^LOX1^–^) from healthy adults (*n* = 9) and PMN-MDSCs (CD11b^+^CD14^–^CD15^+^LOX1^+^) from healthy neonates (*n* = 8) and neonates with inflammation (*n* = 8) was detected by flow cytometry. Clinical parameters are listed in Supplemental Table 1. (**E**) The expression of ODC1 and eIF5A^Hyp^ in neutrophils from healthy adults and PMN-MDSCs from healthy neonates and neonates with inflammation was detected by immunofluorescence (*n* = 3 per group; 3 fields of each donor were randomly selected for calculating the mean fluorescence intensity [MFI]). Clinical parameters are listed in Supplemental Table 1. (**F**) Transcriptional profiling of CD11b^+^Ly6C^lo/–^Ly6G^+^ cells isolated from the spleen of adult mice (AM) and newborn mice (NBM). Heatmaps depict differentially expressed genes involved in polyamine metabolism and transport. (**G** and **H**) The expression of ODC1 in CD11b^+^Ly6C^lo/–^Ly6G^+^ cells was detected by flow cytometry (**G**) (*n* = 4 per group) and immunofluorescence (**H**) (*n* = 3 per group; 3 fields of each donor were randomly selected for calculating the MFI). (**I**) Cellular polyamines in CD11b^+^Ly6C^lo/–^Ly6G^+^ cells were detected by UPLC-MS/MS (*n* = 4 per group). Data are shown as mean ± SEM. Statistical analysis was performed using 1-way ANOVA with Tukey’s multiple-comparison test (**B**–**E**) and unpaired 2-tailed Student’s *t* test (**G**–**I**). Scale bars: 5 μm. Original magnification, ×2.5 (insets, **E** and **H**). *P < 0.05, **P < 0.01, ***P < 0.001.

We next profiled the expression of key enzymes involved in polyamine metabolism, including ornithine decarboxylase 1 (ODC1) and its downstream deoxyhypusine synthase (DHPS) (Figure 3A). Flow cytometric analysis revealed that PMN-MDSCs from healthy infants exhibited markedly higher levels of ODC1 and DHPS (Figure 3, B and C) than did those from infants with inflammation or neutrophils from healthy adults. DHPS facilitates the conversion of eukaryotic translation initiation factor 5A (eIF5A) into physiologically active hypusinated eIF5A (eIF5A^Hyp^), with the ratio of eIF5A^Hyp^ to eIF5A serving as an indicator of the hypusination rate. Consistent with the upregulation of DHPS, the levels of eIF5A^Hyp^, as well as the ratio of eIF5A^Hyp^ to eIF5A, were elevated in PMN-MDSCs from healthy infants (Figure 3D and Supplemental Figure 3, G and H). The elevation of ODC1 and eIF5A^Hyp^ in PMN-MDSCs from healthy infants was further confirmed via immunofluorescence staining (Figure 3E).

In line with the observations from humans, polyamine metabolism was also active in myeloid cells from neonatal mice. Transcriptional profiling revealed substantial upregulation of genes related to polyamine metabolism (*Odc1*, *Srm*, and *Sms*) and polyamine transporters (*Slc3a2*, *Slc18b1*, and *Slc22a3*) in neonatal PMN-MDSCs (CD11b^+^Ly6C^lo/–^Ly6G^+^ cells) compared with the corresponding neutrophils from adult mice (Figure 3F). Flow cytometric analysis (Figure 3G) and immunofluorescence staining (Figure 3H) further confirmed the upregulation of ODC1 in neonatal PMN-MDSCs. In accordance with the upregulation of these key enzymes, the concentrations of putrescine and spermidine were substantially higher in neonatal PMN-MDSCs than in the corresponding neutrophils from adult controls, as determined by UPLC-MS/MS (Figure 3I). Taken together, these observations suggest that polyamine metabolism may play a role in the functionality of PMN-MDSCs during early infancy.

### Polyamine-eIF5A^Hyp^ enhances the immunosuppressive activity of PMN-MDSCs in neonates.

**Figure 4:**
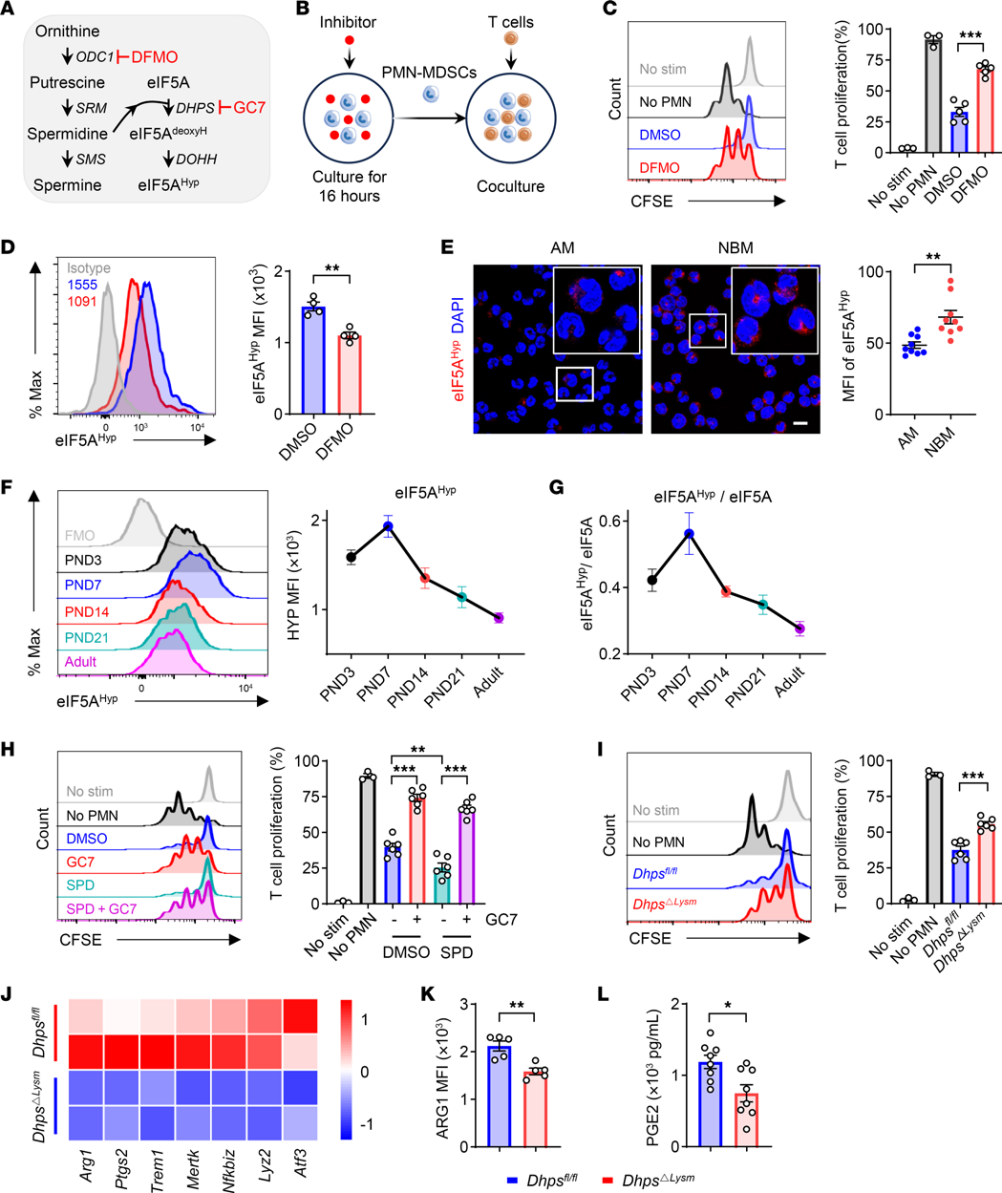
Polyamine-eIF5A^Hyp^ enhances the immunosuppressive activity of PMN-MDSCs in neonates. (**A** and **B**) Pharmacological inhibition of polyamine metabolism (**A**) and coculture strategy (**B**). (**C** and **D**) PMN-MDSCs were treated with DFMO (200 μM) for 16 hours. Suppression of T cell proliferation by PMN-MDSCs (**C**) (*n* = 5 per group) and expression of eIF5A^Hyp^ (**D**) (*n* = 4 per group). Data are representative of 3 independent experiments. (**E**) CD11b^+^Ly6C^lo/–^Ly6G^+^ cells were isolated from adult mice (AM) and newborn mice (NBM). The expression of eIF5A^Hyp^ was detected by immunofluorescence (*n* = 3 per group; 3 fields of each donor were randomly selected for calculating the MFI). Original magnification, ×2.5. (**F** and **G**) The expression of eIF5A^Hyp^ (**F**) (*n* = 4 per group) and hypusination rate (**G**) (*n* = 4 per group) in CD11b^+^Ly6C^lo/–^Ly6G^+^ cells of different ages. Data are representative of 3 independent experiments. (**H**) Suppression of T cell proliferation by PMN-MDSCs, which were pretreated with DMSO, spermidine (SPD) (0.2 μM), GC7 (10 μM), and SPD plus GC7 for 16 hours (*n* = 6 per group). Data are representative of 3 independent experiments. (**I**) Suppression of T cell proliferation by *Dhps^fl/fl^* and *Dhps*^ΔLysm^ PMN-MDSCs (*n* = 6 per group). Data are representative of 3 independent experiments. (**J**) Bulk RNA sequencing of PMN-MDSCs from the spleen of *Dhps^fl/fl^* and *Dhps*^ΔLysm^ littermates. Heatmaps of differentially expressed genes involved in immunosuppressive function. (**K** and **L**) ARG1 (*n* = 5 per group) and cellular PGE_2_ production (*n* = 8 per group) in *Dhps^fl/fl^* and *Dhps*^ΔLysm^ PMN-MDSCs. Data are shown as mean ± SEM. Statistical analysis was performed using 1-way ANOVA with Tukey’s multiple-comparison test (**C** and **I**), unpaired 2-tailed Student’s *t* test (**D**, **E**, **K**, and **L**), and 2-way ANOVA with Šidák’s multiple-comparison test (**H**). Scale bar: 5 μm. *P < 0.05, **P < 0.01, ***P < 0.001.

To evaluate the effect of polyamines on the immunosuppressive function of neonatal PMN-MDSCs, the ODC1 inhibitor α-difluoromethylornithine (DFMO) was used in MDSC–T cell coculture experiments (Figure 4, A and B). Results showed that DFMO pretreatment almost completely abolished the immunosuppressive function of PMN-MDSCs against antigen-specific T cells (Figure 4C). As expected, DFMO reduced the levels of eIF5A^Hyp^ (Figure 4D). The abundance of eIF5A^Hyp^ in neonatal PMN-MDSCs was further confirmed by immunofluorescence staining (Figure 4E). Interestingly, the levels of eIF5A^Hyp^ and the hypusination rate (eIF5A^Hyp^/eIF5A) in PMN-MDSCs were age dependent, peaked at postnatal day 7, and declined thereafter (Figure 4, F and G, and Supplemental Figure 4A). This finding is highly similar to the dynamic appearance of the suppressive activity of PMN-MDSCs in neonates, as we have previously reported (7). The administration of the DHPS inhibitor GC7 effectively eliminated the immunosuppressive activity of PMN-MDSCs, regardless of the presence or absence of spermidine (Figure 4H). In addition, spermidine increased the frequency of PMN-MDSCs and enhanced their immunosuppressive function in vivo (Supplemental Figure 4, B and C). PMN-MDSCs treated with spermidine also exhibited increased bacterial-killing capability (Supplemental Figure 4D). These observations indicate that spermidine enhances the protective functions of PMN-MDSCs in neonates.

For further confirmation, mice with myeloid-specific depletion of *Dhps* (*Dhps^ΔLysm^*) were generated by crossing of *Dhps^fl/fl^* strains with *Lysm^cre^* strains. Although the proportion of PMN-MDSCs was slightly affected after genetic ablation of *Dhps* (Supplemental Figure 4E), the immunosuppressive function of PMN-MDSCs was significantly impaired in *Dhps^ΔLysm^* pups compared with *Dhps^fl/fl^* littermates (Figure 4I). Bulk RNA sequencing analysis revealed that PMN-MDSCs from *Dhps^ΔLysm^* pups expressed lower levels of immunosuppressive genes (*Arg1*, *Ptgs2*, *Atf3*, *Trem1*, etc.) than those from *Dhps^fl/fl^* littermates (Figure 4J). Consistently, *Dhps^ΔLysm^* PMN-MDSCs exhibited lower levels of arginase 1 (ARG1) (Figure 4K) and reduced cellular prostaglandin E_2_ (PGE_2_) production (Figure 4L), both of which are key immunosuppressive mediators of PMN-MDSCs. GC7 administration also impaired the immunosuppressive function of PMN-MDSCs from human infants (Supplemental Figure 4, F and G). These findings underscore the critical role of the spermidine-eIF5A^Hyp^ axis in the immunosuppressive function of neonatal PMN-MDSCs.

### eIF5A^Hyp^ maintains the mitochondrial function of neonatal PMN-MDSCs by modulating the translation of NFU1.

**Figure 5:**
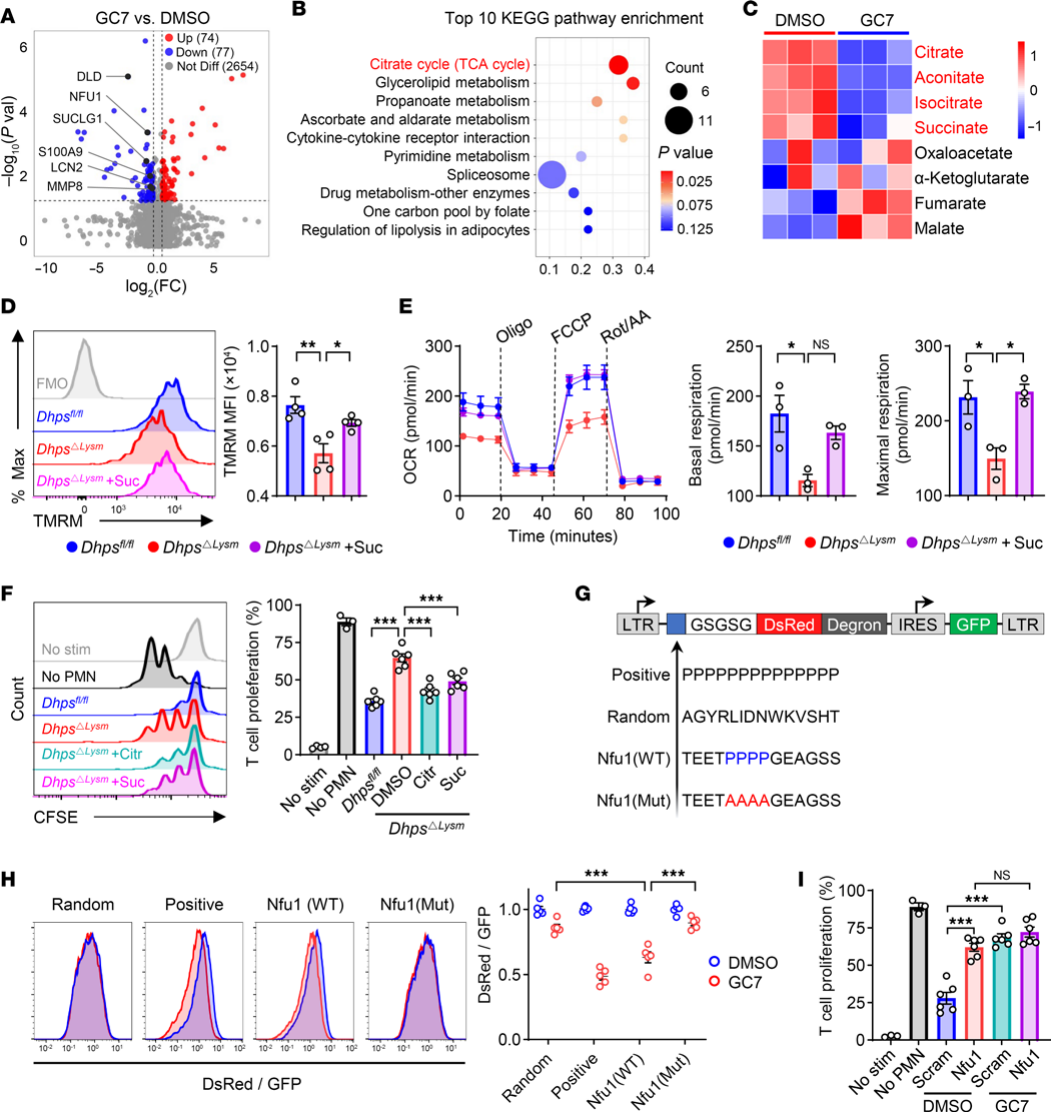
eIF5A^Hyp^ maintains the mitochondrial function of neonatal PMN-MDSCs by modulating translation of NFU1. (**A** and **B**) After treatment with GC7 (10 μM) for 16 hours, PMN-MDSCs were collected for proteomics analysis using LC-MS/MS. Volcano plot of all proteins (**A**) and KEGG analysis (**B**). (**C**) After treatment with GC7 (10 μM) for 16 hours, PMN-MDSCs were collected for targeted metabolomics. (**D**) Mitochondrial membrane potential of *Dhps^fl/fl^* and *Dhps*^ΔLysm^ PMN-MDSCs treated with or without succinate (Suc; 1 μM) was evaluated with tetramethylrhodamine methyl ester (TMRM) by flow cytometry (*n* = 4 per group). Data are representative of 3 independent experiments. (**E**) Oxygen consumption rate (OCR) of *Dhps^fl/fl^* and *Dhps*^ΔLysm^ PMN-MDSCs treated with or without succinate was evaluated by Seahorse assay (*n* = 3 per group). (**F**) Suppression of T cell proliferation by *Dhps^fl/fl^* and *Dhps*^ΔLysm^ PMN-MDSCs, which were pretreated with citrate (Citr; 1 μM) and succinate (Suc; 1 μM) (*n* = 6 per group). Data are representative of 3 independent experiments. (**G**) Target sequences were inserted into N-terminus of DsRed and separated by a GSGSG flexible linker. Transfection was evaluated by green fluorescent protein (GFP), and translation was quantified as the ratio of DsRed to GFP. (**H**) Transfected NIH 3T3 cells were treated with GC7 (10 μM) for 24 hours. Expression of DsRed and GFP was measured by flow cytometry (*n* = 5 per group). Data are representative of 3 independent experiments. (**I**) Suppression of T cell proliferation by PMN-MDSCs. Nfu1 was silenced by Nfu1-shRNA followed by treatment with GC7 (10 μM) (*n* = 6 per group). Data are representative of 3 independent experiments. Data are shown as mean ± SEM. Statistical analysis was performed using 1-way ANOVA with Tukey’s multiple-comparison test (**D**–**F**) and 2-way ANOVA with Šidák’s multiple-comparison test (**H** and **I**). NS, *P* > 0.05; *P < 0.05, **P < 0.01, ***P < 0.001.

Hypusination of eIF5A regulates the translation of mRNAs that are implicated in various biological processes (21–23). We subsequently evaluated the proteomic profile of PMN-MDSCs treated with GC7 or vehicle via liquid chromatography coupled to tandem mass spectrometry (LC-MS/MS). Results revealed that a total of 77 proteins were downregulated and 74 proteins were upregulated after GC7 treatment (Figure 5A). Notably, the expression of immunosuppression-related proteins, such as S100A9, LCN2, and MMP8, was downregulated by GC7 (Figure 5A). Kyoto Encyclopedia of Genes and Genomes (KEGG) enrichment analysis and gene set enrichment analysis showed that the citrate cycle (TCA cycle) was the most significantly altered pathway (Figure 5B and Supplemental Figure 5A). Subsequent analysis of the targeted metabolomics via high-performance liquid chromatography (HPLC)–MS/MS further confirmed a significant reduction in intermediate metabolites from the TCA cycle, including citrate, aconitate, isocitrate, and succinate, in PMN-MDSCs upon GC7 treatment (Figure 5C). These observations suggest that inhibition of DHPS compromises the mitochondrial function of PMN-MDSCs.

Further flow cytometry analysis confirmed that PMN-MDSCs from *Dhps^ΔLysm^* pups exhibited a decreased mitochondrial membrane potential (Figure 5D) and a lower oxygen consumption rate, as measured by the Seahorse assay (Figure 5E). These deficiencies were mitigated by supplementation of succinate (Figure 5, D and E). In line with these observations, spermidine significantly enhanced the basal and maximal mitochondrial respiration of PMN-MDSCs, which was abolished by GC7 coadministration (Supplemental Figure 5B). Similar observations were obtained in neonatal PMN-MDSCs from humans (Supplemental Figure 5C). Importantly, coadministration of the mitochondrial metabolites citrate and succinate efficiently rescued the diminished function of PMN-MDSCs caused by the genetic ablation of *Dhps* (Figure 5F) or GC7 (Supplemental Figure 5D). Considering that CD11b^+^Ly6C^lo/–^Ly6G^+^ cells sorted by flow cytometry may capture both polymorphonuclear cells and PMN-MDSCs, density gradient separation was performed followed by gating of CD11b^+^Ly6C^lo/–^Ly6G^+^ cells, to purify PMN-MDSCs (Supplemental Figure 5E). Consistent results were obtained under both GC7 treatment (Supplemental Figure 5F) and genetic ablation of *Dhps* (Supplemental Figure 5G).

The downstream events of polyamine metabolism involve the hypusination of eIF5A, which facilitates translation elongation by enhancing peptide bond formation in specific peptide sequences, such as polyproline sequences (24). Thus, we propose that eIF5A^Hyp^ regulates the translation of certain mitochondrial protein(s) that contain polyproline sequences. Among the differentially expressed mitochondrial proteins that were identified via proteomic profiling (Figure 5A), NFU1 (iron-sulfur cluster scaffold) is a potential target for translational regulation by hypusine based on the presence of polyproline sequences. After being translated in the cytoplasm, NFU1 is transported to the mitochondria, where it functions as a carrier of iron-sulfur clusters, thus aiding in the transfer of 4Fe-4S clusters to respiratory chain complexes I and II, and lipoyl synthase. This mechanism plays a crucial role in regulating the electron transport chain, as well as the functions of pyruvate dehydrogenase (PDH) and α-ketoglutarate dehydrogenase (KGDH), thus ultimately impacting oxidative phosphorylation and the TCA cycle (Supplemental Figure 5H) (25, 26). The downregulation of NFU1 protein expression by GC7 was confirmed by flow cytometry (Supplemental Figure 5I), although no change in its mRNA level was detected (Supplemental Figure 5J). This finding raises the possibility that the regulation of NFU1 by GC7 may occur at the protein level. The polyproline sequence of NFU1 (whether wild type or mutant) was then inserted into a plasmid with DsRed/GFP tags. The translation of the inserted sequence was subsequently evaluated in NIH 3T3 cells via the DsRed/GFP ratio in the presence or absence of GC7 treatment (Figure 5G). The results indicated that the translation of the polyproline sequence was decreased following GC7 treatment, whereas the mutation of polyproline to polyalanine abolished this effect (Figure 5H), suggesting that eIF5A^Hyp^ is necessary for the efficient translation of the polyproline motif of NFU1. Random and positive sequences were evaluated in parallel (Figure 5, G and H). These findings provide evidence that NFU1 is a direct target of eIF5A^Hyp^.

Next, Nfu1 expression was knocked down via lentivirus transduction of shRNA when bone marrow cells were cultured to induce the differentiation of MDSCs in vitro. The downregulation of NFU1 was confirmed via quantitative real-time PCR and flow cytometry (Supplemental Figure 5, K and L). The immunosuppressive activity of PMN-MDSCs was significantly compromised in the Nfu1-shRNA–transduced group, and the administration of GC7 failed to display noticeable effects after Nfu1 knockdown (Figure 5I). Additionally, the levels of ARG1 expression and PGE_2_ production in PMN-MDSCs, as well as mitochondrial potential of PMN-MDSCs, exhibited consistent changes (Supplemental Figure 5, M–O). These observations suggest that eIF5A^Hyp^-mediated NFU1 translation contributes to the mitochondrial function and subsequent immunosuppressive activity of neonatal PMN-MDSCs.

### Histone acetylation–mediated epigenetic regulation contributes to the effect of eIF5A^Hyp^ on PMN-MDSCs.

**Figure 6:**
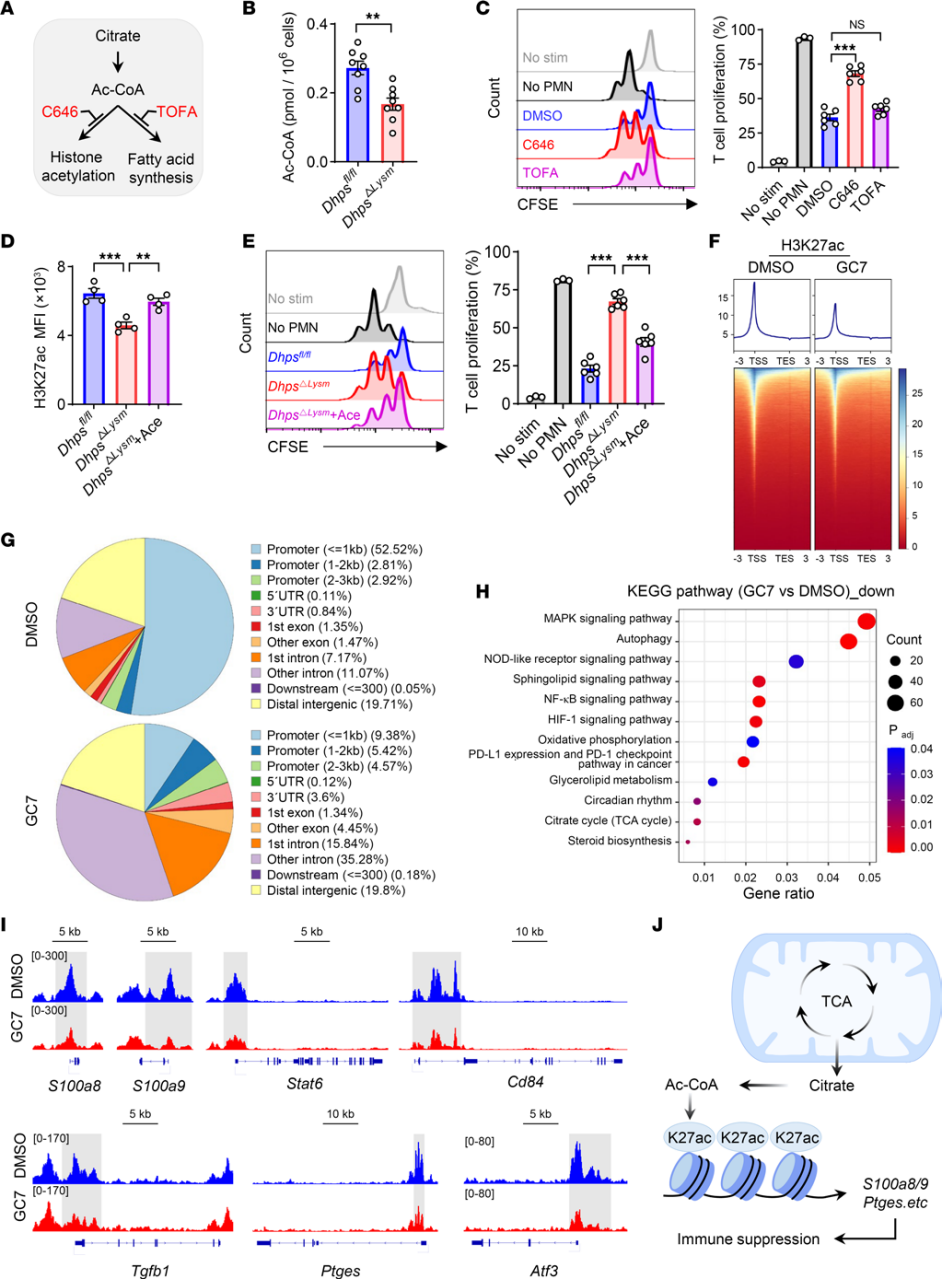
Histone acetylation–mediated epigenetic regulation contributes to the effect of eIF5A^Hyp^ on PMN-MDSCs. (**A**) Pharmacological inhibition of acetyl-coenzyme A (Ac-CoA) metabolic pathway. (**B**) Cellular Ac-CoA was detected in *Dhps^fl/fl^* and *Dhps*^ΔLysm^ PMN-MDSCs (*n* = 8 per group). (**C**) Suppression of T cell proliferation by PMN-MDSCs, which were pretreated with C646 (10 μM) and TOFA (5 μM) (*n* = 6 per group). Data are representative of 3 independent experiments. (**D**) The expression of H3K27ac in *Dhps^fl/fl^* and *Dhps*^ΔLysm^ PMN-MDSCs treated with or without acetate (Ace; 1 μM) (*n* = 4 per group). Data are representative of 3 independent experiments. (**E**) Suppression of T cell proliferation by *Dhps^fl/fl^* and *Dhps*^ΔLysm^ PMN-MDSCs, which were pretreated with or without acetate (Ace; 1 μM) (*n* = 6 per group). Data are representative of 3 independent experiments. (**F**–**I**) After treatment with GC7 (10 μM), CUT&Tag was performed using an antibody against H3K27ac. (**F**) Heatmaps of coverage around the H3K27ac peaks (within 3 kb of transcription start site [TSS] and transcription end site [TES]). (**G**) Levels of H3K27ac in different regions. (**H**) KEGG enrichment for downregulated genes. (**I**) H3K27ac coverage at genes by Integrative Genomics Viewer. (**J**) Schematic model of mitochondrial metabolites for regulating the function of PMN-MDSCs. Data are shown as mean ± SEM. Statistical analysis was performed using unpaired 2-tailed Student’s *t* test (**B**) and 1-way ANOVA with Tukey’s multiple-comparison test (**C**–**E**). NS, *P* > 0.05; *P < 0.05, **P < 0.01, ***P < 0.001.

We next explored the downstream events of rewired mitochondrial function induced by the polyamine-eIF5A^Hyp^ axis. Mitochondria-derived intermediate metabolites are important for epigenetic gene regulation (27). Citrate is converted to acetyl-coenzyme A (acetyl-CoA) by ATP citrate lyase following its export from the mitochondria, thereby serving as a substrate for histone acetylation–mediated epigenetic regulation or fatty acid synthesis (Figure 6A) (28). The amount of cellular acetyl-CoA in PMN-MDSCs was significantly reduced after the genetic ablation of *Dhps* (Figure 6B) or GC7 treatment (Supplemental Figure 6A). The administration of the histone acetylation inhibitor C646 effectively diminished the immunosuppressive function of PMN-MDSCs, whereas the fatty acid synthesis inhibitor TOFA displayed a minimal effect (Figure 6C). Additionally, the level of histone H3 lysine 27 acetylation (H3K27ac) in PMN-MDSCs was reduced via the genetic ablation of *Dhps* or GC7, which was rescued by the coadministration of acetate (Figure 6D and Supplemental Figure 6B). Moreover, acetate administration restored the diminished immunosuppressive activity caused by the genetic ablation of *Dhps* or GC7 (Figure 6E and Supplemental Figure 6C). These findings suggest that histone acetylation is the primary downstream pathway for acetyl-CoA utilization in PMN-MDSCs.

Subsequently, H3K27ac binding profiling in PMN-MDSCs in response to GC7 treatment was evaluated via CUT&Tag assay. The results revealed substantial alterations in the global levels of H3K27ac in PMN-MDSCs treated with GC7 (Figure 6F). A total of 5,326 peaks decreased, whereas 1,871 peaks increased, following GC7 treatment (Supplemental Figure 6D). Notably, the enrichment of H3K27ac was primarily observed at promoter regions, which was dramatically diminished by GC7 treatment (Figure 6G). Pathway enrichment analysis revealed a significant downregulation of signaling pathways associated with immunosuppressive activity, including MAPK signaling, NF-κB signaling, HIF-1 signaling, and the TCA cycle, following treatment with GC7 (Figure 6H). Furthermore, GC7 treatment resulted in decreased levels of H3K27ac in immunosuppressive genes such as *S100a8*, *S100a9*, *Stat6*, *Cd84*, *Tgfb1*, *Ptges*, *Atf3*, etc. (Figure 6I and Supplemental Figure 6E). These findings suggest that the impact of eIF5A^Hyp^ on PMN-MDSCs is mediated by histone acetylation–driven epigenetic processes. More specifically, metabolites derived from the TCA cycle promote histone acetylation, thereby facilitating the epigenetic control of genes linked to immunosuppression. This axis plays an important role in the immunosuppressive activity of PMN-MDSCs in neonates (Figure 6J).

### Spermidine attenuates neonatal inflammation through eIF5A^Hyp^ in PMN-MDSCs.

**Figure 7:**
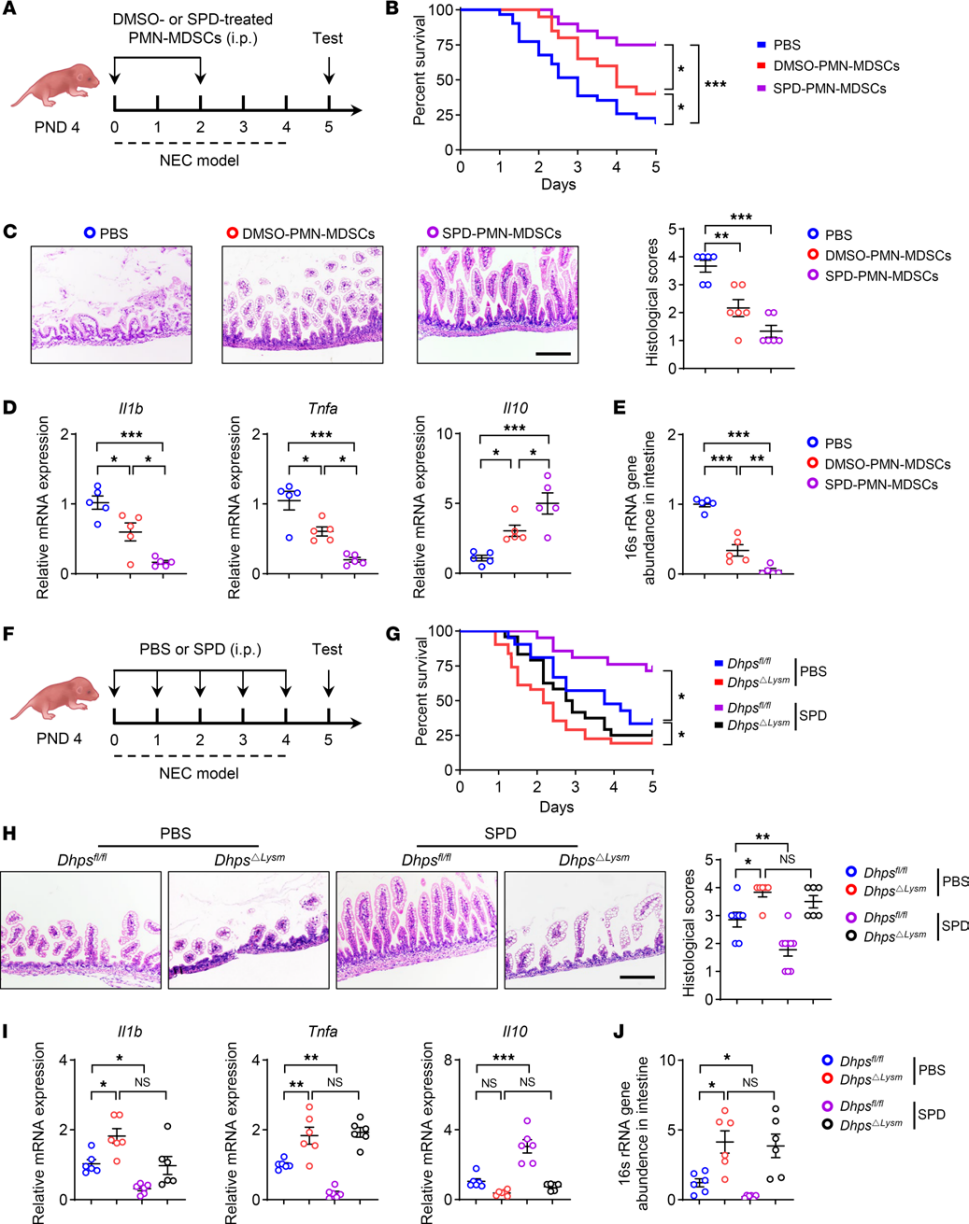
Spermidine attenuates neonatal inflammation through eIF5A^Hyp^ in PMN-MDSCs. (**A**) Schematic approach for transfer of PMN-MDSCs during NEC induction. PND, postnatal day. (**B**) Survival curve after induction of NEC (PBS, *n* = 31; DMSO-PMN-MDSCs, *n* = 20; SPD-PMN-MDSCs, *n* = 20). Data combined from 2 independent experiments. (**C**) H&E staining and histopathological scores of intestine tissues (*n* = 6 per group). (**D**) Relative mRNA expression of *Il1b*, *Tnfa*, and *Il10* was determined by quantitative real-time PCR (*n* = 5 per group). (**E**) Bacterial abundance in the intestinal wall was evaluated by 16S abundance (*n* = 5 per group). (**F**) Schematic approach for treatment with spermidine (SPD) (10 mg/kg/d). (**G**) Survival curve after induction of NEC (*Dhps^fl/fl^*, *n* = 21; *Dhps*^ΔLysm^, *n* = 31; *Dhps^fl/fl^* + SPD, *n* = 21; *Dhps*^ΔLysm^ + SPD, *n* = 24). Data combined from 2 independent experiments. (**H**) H&E staining and histopathological scores of intestinal tissues (*Dhps^fl/fl^*, *n* = 7; *Dhps*^ΔLysm^, *n* = 6; *Dhps^fl/fl^* + SPD, *n* = 9; *Dhps*^ΔLysm^ + SPD, *n* = 6). (**I** and **J**) Relative mRNA expression of *Il1b*, *Tnfa*, and *Il10* (**I**) (*n* = 6 per group) and bacterial abundance in the intestinal wall (**J**) (*n* = 6 per group). Data are shown as mean ± SEM. Statistical analysis was performed using log-rank (Mantel-Cox) test (**B** and **G**), 1-way ANOVA with Tukey’s multiple-comparison test (**C**–**E**), and 2-way ANOVA with Šidák’s multiple-comparison test (**H**–**J**). Scale bars: 100 μm. NS, *P* > 0.05; *P < 0.05, **P < 0.01, ***P < 0.001.

We investigated the role of eIF5A hypusination–mediated PMN-MDSCs in the therapeutic efficacy of spermidine in neonatal inflammation. Neonatal PMN-MDSCs were exposed to spermidine to augment their immunosuppressive activity, and then transferred into neonatal mice during the course of NEC induction (Figure 7A). PMN-MDSCs exposed to spermidine exhibited enhanced therapeutic efficacy in mitigating the severity of NEC, as evidenced by increased survival rates, decreased intestinal inflammation, reduced expression of proinflammatory genes (*Il1b* and *Tnfa*), upregulated expression of an antiinflammatory gene (*Il10*), and decreased bacterial abundance in the intestinal wall (Figure 7, B–E). Furthermore, the transfer of spermidine-treated PMN-MDSCs after NEC induction also caused the remission of NEC (Supplemental Figure 7, A–E). Given the importance of the spermidine-eIF5A^Hyp^ metabolic axis in modulating the immunosuppressive activities of PMN-MDSCs, we postulated that spermidine may mitigate inflammation through the hypusination of eIF5A. *Dhps^ΔLysm^* pups and *Dhps^fl/fl^* littermates were supplemented with spermidine during NEC induction (Figure 7F). In accordance with the impaired function of PMN-MDSCs, *Dhps^ΔLysm^* pups exhibited exacerbated severity of NEC compared with *Dhps^fl/fl^* controls, which was characterized by decreased survival rates, heightened intestinal inflammation, elevated expression of proinflammatory genes (*Il1b* and *Tnfa*), downregulated expression of an antiinflammatory gene (*Il10*), and increased bacterial abundance in the intestinal wall (Figure 7, G–J). Nevertheless, the administration of spermidine led to notable alleviation of NEC symptoms in *Dhps^fl/fl^* littermates, but not in *Dhps^ΔLysm^* pups (Figure 7, G–J). These findings suggest that the amelioration of neonatal inflammation by spermidine is dependent on the eIF5A^Hyp^ metabolic axis in myeloid cells.

## Discussion

This study demonstrates the role of polyamines in modulating the immunosuppressive activity of PMN-MDSCs and underscores the potential therapeutic significance of spermidine in managing neonatal inflammation. Spermidine governs the translation of NFU1, a critical determinant of mitochondrial function, which elicits a metabolic-epigenetic network in neonatal PMN-MDSCs.

Polyamines provide numerous health benefits and can be acquired through the intestinal microbiota, dietary intake, and cellular metabolism (15). Breast milk contains abundant polyamines, with levels peaking during the first week postpartum, followed by a gradual decline throughout lactation (29). In addition, maternal consumption of polyamine-rich foods, such as vegetables and fruits, was linked to increased polyamine levels in breast milk (30). Polyamines in breast milk may affect gut development and the immune system in early infancy, whereas the specific role of polyamines in neonates is not fully understood (17). After birth, neonates transit from the sterile intrauterine environment to the microorganism-rich extrauterine environment. During this critical early postnatal period, the immune system of newborns is not fully developed, rendering them more susceptible to inflammation as a result of microbial colonization or pathogen invasion (3). In contrast, adults have a fully developed immune system that is adapted to coexist with microbiota and effectively defend against potential pathogens. We have previously demonstrated that neonatal MDSCs display strong immunosuppressive function, as well as antibacterial function, which contribute to the control of inflammation in early life (7, 8). In this study, we demonstrate that the elevation of polyamines in newborns represents a protective mechanism that mitigates potential inflammation following exposure to substantial quantities of microorganisms immediately after birth, in which PMN-MDSCs participate.

Polyamines have been shown to have diverse immunoregulatory effects on different types of immune cells. Polyamines play a role in the differentiation of T cells through epigenetic modifications, and regulate autophagy in plasma B cells (23, 31). Moreover, supplementation with spermidine has been shown to enhance the antitumor activity of CD8^+^ T cells by improving mitochondrial function (32). In myeloid cells, polyamines promote M2 macrophage polarization and enhance the antimicrobial ability of macrophages (22, 33). Miska et al. reported that inhibition of the synthesis of polyamines could reduce the population of immunosuppressive tumor-associated myeloid cells and enhance the antitumor response by decreasing the intracellular pH (34). This study reveals that polyamines play a role in maintaining the immunosuppressive properties of neonatal PMN-MDSCs through a mitochondria-mediated metabolic-epigenetic network. We highlight the importance of polyamines in the control of neonatal inflammation, in which immunosuppressive PMN-MDSCs are involved. eIF5A^Hyp^-mediated translation of NFU1 and mitochondrial function represents the underlying mechanism.

Among the polyamines, spermidine is the exclusive substrate for the hypusination of eIF5A, which facilitates the translation of peptides containing specific sequences, including the polyproline motif (21–24, 35). Puleston et al. reported that mitochondrial targeting sequences (MTSs) are specific sequences regulated by eIF5A^Hyp^ (22). The inhibition of hypusine fundamentally downregulates the expression of mitochondrial proteins, such as succinate dehydrogenase complex flavoprotein subunit A (SDHA), methylmalonyl-CoA mutase (MCM), and succinyl-CoA synthetase (SUCLG1). These proteins contain MTSs and are directly regulated by eIF5A^Hyp^ at the translational level. Our proteomic profiles revealed that several mitochondrial proteins, such as NFU1, SUCLG1, and dihydrolipoamide dehydrogenase (DLD), were downregulated after the blockage of DHPS, indicating the importance of eIF5A^Hyp^ in mitochondrial function. In this study, we focused on NFU1 as a direct target of eIF5A^Hyp^ because it contains a polyproline motif and because of its broad significance in multiple mitochondrial proteins, such as respiratory chain complexes I and II, lipoic acid, PDH, and KGDH (25, 26). We confirmed that the translation of NFU1 was regulated by polyamines, which contributes to the immunosuppressive function of neonatal PMN-MDSCs.

Numerous studies have shown that spermidine supplementation mitigates inflammation in adults, primarily by facilitating the polarization of antiinflammatory macrophages, preserving epithelial barrier integrity, and inhibiting inflammatory dendritic cells (36–38). Notably, administration of spermidine in neonatal endotoxemia and NEC mouse models led to a significant reduction in inflammatory responses and favorable outcomes, indicating a therapeutic role of spermidine in treating neonatal inflammatory disorders. Furthermore, long-term benefits, including enhanced weight gain, were observed following treatment with spermidine-treated PMN-MDSCs in the mouse model (data not shown). From the perspective of clinical translation, spermidine could be used for prevention in neonates who are at high risk of inflammatory disorders, such as those with extremely low birth weights, asphyxia, and so on. For patients diagnosed with neonatal inflammatory disorders, spermidine could be used to attenuate inflammatory responses and may improve the survival rate. However, the safety and efficacy of spermidine in human neonates need to be confirmed in future studies.

There are several limitations in this study. First, regarding the translatability of the preclinical model work to the human model: Assessing the correlation between PMN-MDSCs and spermidine in clinical samples could substantiate the significance of spermine–PMN-MDSC dysregulation in the pathogenesis of neonatal inflammation. However, owing to the challenges of obtaining the necessary samples, we were unable to perform this correlation analysis. Second, the potential impacts of polyamines on other immune cells in early infancy deserve further investigations. Third, it was found that higher levels of plasma spermidine at birth were correlated with lower susceptibility to inflammatory disorders. Future study should involve a larger sample size of infants and incorporate multicenter studies; predictive modeling should also be employed before definitive conclusions are drawn regarding the predictive value of spermidine in neonatal inflammation.

## Methods

### Sex as a biological variable.

Sex was not considered as a biological variable. Both sexes were used for human and mouse studies.

### Animals.

Mice were maintained in a specific pathogen–free environment with a 12-hour light/12-hour dark cycle. Animals were on a C57BL/6J genetic background. *Dhps^fl/fl^* and wild-type mice were obtained from Cyagen Bioscience Inc., while *Lysm^cre^* mice and OT-I mice were obtained from The Jackson Laboratory. *Dhps^fl/fl^ Lysm^cre^* (*Dhps^ΔLysm^*) mice were generated by crossing of *Dhps^fl/fl^* strains with *Lysm^cre^* strains.

### Animal models.

The NEC model was established as previously described (8, 39). Briefly, 4-day-old mice were subjected to the combination of formula feeding, hypoxia (5% O_2_), hypothermia, and the oral gavage of enteric bacteria. Mice were treated with spermidine (10 mg/kg/d) by intraperitoneal (i.p.) injection or oral gavage during the induction of NEC. For the adoptive transfer experiment, 1.5 × 10^6^ DMSO- or spermidine-treated PMN-MDSCs were injected i.p. 1 hour before the NEC procedure on days 0 and 2. Mice were sacrificed at day 5, and intestine was collected for H&E staining and quantitative real-time PCR. For post-treatment of the NEC model, survived NEC mice were treated with spermidine (10 mg/kg/d, i.p. injection) for 5 days; adoptive transfer of PMN-MDSCs was performed on days 5 and 7 after NEC induction. Mice were sacrificed at day 10, and intestine was collected for H&E staining and quantitative real-time PCR.

Neonatal endotoxemia was induced by i.p. injection of LPS (15 mg/kg). For administration of spermidine, 4-day-old mice were subjected to spermidine (10 mg/kg/d, i.p. injection) for 5 days before induction of endotoxemia. For post-treatment of neonatal endotoxemia, 8-day-old mice were treated with spermidine (10 mg/kg, i.p. injection) 2 hours after i.p. injection of LPS (15 mg/kg). Mice were sacrificed at 24 hours, and lung, liver, and kidney were collected for H&E staining and quantitative real-time PCR.

### Immune cell isolation and treatment.

For in vitro experimentation (8), PMN-MDSCs from spleen of 7- to 8-day-old mice were isolated, followed by culturing in 1640 medium (20 ng/mL mouse recombinant GM-CSF, 10% FBS, and 1% penicillin-streptomycin mixed) with indicated chemicals or inhibitors.

Isolation of immune cells from intestinal lamina propria was performed as previously described (39). Briefly, small intestine was opened longitudinally and washed with cold PBS, followed by cutting into pieces. Epithelial cells were removed by shaking in HBSS (containing 1 mM DTT, 2 mM EDTA, 10 mM HEPES, and 5% FBS) for 20 minutes. The remaining tissues were digested with 0.5 mg/mL collagenase I (Invitrogen) and 1 ng/mL DNase I (Solarbio) for 50 minutes. Leukocytes were enriched by Percoll (Cytiva) gradient centrifugation (40% and 80%). Single-cell suspensions were used for flow cytometry staining.

### Immunosuppressive function of PMN-MDSCs.

The assessment of PMN-MDSC–mediated suppression was conducted as previously described (8, 39). Specifically, CD8^+^ OT-I T cells were mixed with splenocytes from WT mice at a 1:4 ratio and labeled with CFSE. These labeled cells were then cocultured with PMN-MDSCs in 1640 medium supplemented with cognate peptides (SIINFEKL, 0.5 ng/mL). Negative controls consisted of splenocytes that incubated without PMN-MDSCs or cognate peptides, while positive controls incubated solely with cognate peptides in the absence of PMN-MDSCs.

### Antimicrobial activity of PMN-MDSCs.

Antimicrobial activity was conducted as previously described (39). Briefly, a total of 2 × 10^5^ PMN-MDSCs were incubated with *Escherichia coli* or *Cronobacter sakazakii* at 37°C and agitated for 1 hour. Then, bacterial solution underwent a series of dilutions and was seeded on culture plates for 14–16 hours. Antibacterial activity was evaluated by calculation of the colony-forming units (CFU) in the last dilution.

### Human samples.

In cohort 1, human plasma or whole-blood samples were collected from newborns with or without inflammatory disorders in Guangzhou Women and Children’s Medical Center (Guangzhou, China) and Tianjin Children’s Hospital (Tianjin, China), and clinical information is listed in Supplemental Table 1. In cohort 2, plasma samples were collected from healthy neonates within 2 days of birth in the Children’s Hospital of Fudan University (Shanghai, China), and clinical information is listed in Supplemental Table 2. Isolation of human PMN-MDSCs and neutrophils from adults was performed as previously described (40). Peripheral-blood samples were collected using EDTA-containing tubes and centrifuged at 800*g* for 5 minutes. The pellets were suspended with normal saline and gently added to the top layer of Lymphoprep (Serumwerk Bernburg AG), followed by centrifugation at 300*g* for 30 minutes with slow acceleration and brakes turned off. The mononuclear cell fraction (low density) was collected for isolation of PMN-MDSCs (CD11b^+^CD14^–^CD15^+^LOX1^+^) in neonatal samples, and sediment (high density) was collected for isolation of neutrophils (CD11b^+^CD14^–^CD15^+^LOX1^–^) in adult samples (Supplemental Figure 8).

Definitions of neonatal diseases are listed below. Respiratory distress syndrome was defined as increasing respiratory distress, requirement of increasing concentrations of oxygen, or need of ventilatory support from the first 6 hours of life with a chest radiograph showing generalized reticular granular pattern with or without an air bronchogram (41). Sepsis was defined by clinical signs and symptoms or culture-proven sepsis (42). NEC was defined by Bell’s staging criteria (stage 2 or 3); the stages were based on systemic signs, intestinal signs, and radiological signs (43). Diagnosis of pneumonia was based on the combination of clinical presentation, radiographic evidence, and laboratory data. Urinary tract infection was defined as positive urine culture with at least 10,000 CFU/mL (44).

### Flow cytometry analysis and sorting.

For cytometry analysis, single-cell suspensions were stained with LIVE/DEAD Fixable Dead Cell Stain Kits to exclude dead cells. The cells were stained with surface marker with a cocktail of fluorescently labeled antibodies at 4°C for 30 minutes. For intracellular staining, cells were stained with surface marker antibodies, followed by fixation and permeabilization using Intracellular Fixation & Permeabilization Buffer. Cells were then incubated with rabbit anti-hypusine (1:200), rabbit anti-EIF5A (1:200), rabbit anti-ODC1 (1:200), rabbit anti-DHPS (1:200), rabbit anti-NFU1 (1:200), and rabbit anti-H2K27ac (1:200) primary antibodies at room temperature for 50 minutes, followed by incubation with secondary antibodies (donkey anti-rabbit Alexa Flour 647, 1:600) for 30 minutes. Data were analyzed using FlowJo software (v10).

For cytometric sorting, PMN-MDSCs were isolated from spleens of 7-day-old mice, while OT-I CD8^+^ T cells were isolated from spleens of 6- to 8-week-old mice. The gating strategy for PMN-MDSCs was CD11b^+^ Ly6C^lo/–^Ly6G^+^ and CD3^+^CD8^+^ for OT-I CD8^+^ T cells. Antibodies are listed in Supplemental Table 3. The strategy of flow cytometry used in this study is provided in Supplemental Figure 8.

### Reagents.

Reagents are listed in Supplemental Table 4.

### Histological analysis of tissues.

Tissues were fixed in formalin and embedded with paraffin. Embedded tissues were sectioned at a thickness of 4 μm and stained with H&E. The severity of intestinal damage in the NEC model was evaluated by a blinded pathologist based on a histological scoring system as previously described (45): grade 0, no damage; grade 1, mild separation of lamina propria and/or submucosa; grade 2, moderate separation of lamina propria and/or submucosa; grade 3, severe separation of lamina propria and/or submucosa or villus sloughing; grade 4, loss of villi and necrosis. The severity of organ damage in the endotoxemia model was assessed by a blinded pathologist using a scoring system as outlined in previous studies (46). Tissue damage in lung and liver was evaluated by assigning of scores ranging from 0 to 3 for each parameter. Assessment of lung damage included criteria such as alveolar hyperemia, hemorrhage, leukocyte infiltration, and thickness of the alveolar wall. Evaluation of liver damage included assessing necrosis, inflammation, ballooning degeneration, and disruption of hepatic cord structure. Kidney injury was scored on a scale of 0 to 5, based on renal tubule and glomerular injuries.

### Immunofluorescence staining.

Cells were isolated by cytometric sorting and fixed with paraformaldehyde, then blocked with 5% BSA and permeabilized with 0.1% Triton X-100. All samples were incubated with primary antibody at 4°C overnight, followed by incubation with secondary antibody at room temperature for 1 hour and DAPI for 10 minutes. Confocal microscopy was carried out using a Zeiss LSM900. Three donors were included in each group, and 3 fields of each donor were randomly selected to calculate the mean fluorescence intensity using ImageJ (NIH).

### Quantitative real-time PCR.

Total RNA was extracted from intestine, lung, liver, and PMN-MDSCs using Trizol reagent, and then reverse-transcribed into cDNA using the Primescript RT Reagent Kit (Takara). Quantitative real-time PCR was performed in a 10 μL reaction volume, which contained cDNA, specific primers, and SYBR Green Master Mix (GenStar BioSolutions). Expression relative to the *Actb* gene was calculated using the *ΔΔ*Ct method (47). Primer sequences are listed in Supplemental Table 5.

### Seahorse metabolic assay.

PMN-MDSCs were resuspended in DMEM (MilliporeSigma) containing 4.5 g/L glucose, 1 mM pyruvate, and 4 mM glutamine at a pH of 7.4. Cells were then plated onto XF24 cell culture microplates at a density of 0.4 × 10^6^ cells per well and incubated without CO_2_ at 37°C for 30 minutes. Oxygen consumption rates (OCRs) were measured at 5-minute intervals after the sequential addition of 2.5 μg/mL oligomycin, 2 μM fluorocarbonyl cyanide phenylhydrazone (FCCP), and 2 μM rotenone/antimycin A. Basal OCR and maximal OCR were determined by averaging of individual measurements taken before the addition of oligomycin and after the addition of FCCP. Data were analyzed using Seahorse Wave software (v2.6.1, Agilent).

### Plasmid construction, lentivirus production, and infection.

To assess translational regulation by hypusine, the target sequences of NFU1 polyproline motif, 14 consecutive prolines, 14 random amino acids, and a mutant NFU1 (PPPP to AAAA), were cloned into the pHAGE-GPS3.0-DEST vector. These target sequences were fused to the N-terminus of DsRed with a flexible GSGSG linker. Additionally, the C-terminal 37–amino acid degron of murine ODC was fused to reduce the half-life of the DsRed fusion protein. To knock down Nfu1, the shRNA sequence (GAAGAGTTAGACTGGAATTTA) was obtained from the Sigma-Aldrich Mission shRNA Library and cloned into the lentiviral vector pLKO.1.

For lentivirus production, 293T cells (American Type Culture Collection) were transfected with the target plasmid and lentiviral packaging plasmids (pMD2.G and psPAXs). The supernatant was collected and then filtered using a 0.45 μm filter. For pHAGE-lentivirus infection, NIH 3T3 cells (Procell Life Science & Technology Co.) were cultured with lentiviral supernatants (containing 10 μg/mL Polybrene), followed by centrifugation (900*g* for 45 minutes). Hygromycin was used to eliminate non-infectious cells after 2 days of infection. For shRNA-lentivirus infection (8), bone marrow cells from neonatal mice were cultured overnight before being exposed to lentiviral supernatants (containing 5 μg/mL Polybrene), followed by centrifugation (900*g* for 45 minutes), and subsequently replenished with lentivirus-free RPMI 1640 after 6 hours. Secondary infection was performed the next day.

### ELISA.

A total of 2 × 10^6^ PMN-MDSCs were collected and underwent 3 freeze-thaw cycles prior to sonication. The supernatant was used for the detection of PGE_2_ (MEIMIAN) or acetyl-CoA (Elabscience) according to the manufacturer’s instructions. For the detection of polyamines, plasma was subjected to centrifugation at 15,000*g* for 15 minutes. The supernatant was then transferred to a new tube and centrifuged for an additional 8 minutes. Polyamines were detected according to the manufacturer’s instructions of the Human Putrescine ELISA Kit (MEIMIAN), Human Spermidine ELISA Kit (Mlbio), Human Spermine ELISA Kit (Mlbio) and Mouse Spermidine ELISA Kit (Mlbio).

### Polyamine quantification by ultra-performance liquid chromatography–tandem mass spectrometry (UPLC-MS/MS).

Cell samples were lysed by sonication, while plasma samples were mixed with pre-cooled methanol for protein precipitation. After centrifugation, supernatant was collected and dried by nitrogen followed by redissolution in acetonitrile-water containing formic acid. The solution was mixed with borate buffer containing TCEP and ascorbic acid. After vortex mixing, 5-AIQC solution was then added and incubated at 55 °C for 10 minutes. The mixture was cooled down to the ambient temperature and added with formic acid. The mixture underwent centrifugation, and the resulting supernatant was subsequently filtered through a 0.22 μm membrane filter. The 5-AIQC–tagged samples were injected individually onto an ultra-performance liquid chromatography (UPLC) column (Agilent ZORBAX Eclipse Plus C18 column, 2.1 × 100 mm, 1.8 μm particles). Multiple-reaction monitoring was used for quantification of screening fragment ions. Peak determination and integration of peak areas were carried out using MassHunter Workstation software (Agilent, version B.08.00). Standard curves were generated, and quantification of samples was performed identically.

### Proteomic analysis by nanoscale liquid chromatography coupled to tandem mass spectrometry (LC-MS/MS).

A total of 4 × 10^6^ PMN-MDSCs were treated with GC7 for 16 hours. Cells were lysed and centrifuged at 20,000*g* for 20 minutes. The supernatant was collected and treated with dithiothreitol (DTT; 37°C for 1 hour) followed by treatment with iodoacetamide in the dark for 30 minutes. Protein quantification was carried out using the Bradford method, 150 μg of protein was digested with trypsin, and the peptides underwent desalting using Waters solid-phase extraction cartridges, followed by vacuum drying and redissolution in 0.1% formic acid. The resulting supernatant was then collected and injected into a self-loading C18 column for separation. The separation process was carried out using a Thermo Fisher Scientific EASY-nLC 1200 system operating at a flow rate of 300 nL/min. The separated peptides were ionized via nano-electrospray ionization and subsequently transferred to an Orbitrap Exploris 480 mass spectrometer (Thermo Fisher Scientific) for detection in data-dependent acquisition mode.

### Targeted metabolomics of central carbon metabolism by high-performance liquid chromatography–tandem mass spectrometry (HPLC-MS/MS).

A total of 4 × 10^6^ PMN-MDSCs were treated with GC7 for 16 hours. Cells were resuspended in water, which was mixed with methanol and formic acid, and subjected to sonication. After centrifugation, supernatant was diluted to final concentration containing 53% methanol. HPLC-MS/MS analysis was performed using an ExionLC AD system (SCIEX) coupled with a QTRAP 6500+ mass spectrometer (SCIEX). Samples were injected onto a Waters Atlantis premier BEH C18 Column (2.1 × 100 mm) using a 10-minute linear gradient at a flow rate of 0.3 mL/min. The eluents were eluent A (0.5% formic acid, 30 mM ammonium formate/water) and eluent B (0.5% formic acid/methanol). The detection of the experimental samples using multiple-reaction monitoring was based on a Novogene in-house database. The data files were processed using SCIEX OS version 1.4.

### Bulk RNA sequencing.

A total of 3 × 10^5^ PMN-MDSCs were isolated from neonatal spleen (postnatal day 7), and total RNA was extracted using Trizol reagent. Quality checks for purification and quantification of isolated RNA were evaluated using a NanoDrop ND-1000 spectrophotometer. Sequencing was performed using an Illumina NovaSeq 6000. Raw reads were filtered to get high-quality clean reads using Cutadapt (v1.9) (https://cutadapt.readthedocs.io/en/stable/installation.html). Subsequently, reads were aligned to the mouse reference genome using the HISAT2 (v2.2.1) package (https://daehwankimlab.github.io/hisat2/). StringTie (v2.1.6) (https://ccb.jhu.edu/software/stringtie/) and ballgown (https://github.com/alyssafrazee/ballgown) were used to estimate the expression levels of all transcripts and assess expression abundance of mRNAs by calculating fragments per kilobase per million mapped reads (FPKM).

### Flux balance analysis.

Single-cell transcriptome profiles (Gene Expression Omnibus GSE253963) were used to compute single-cell-level metabolic activity by COMPASS (v0.9.10.2), an algorithm based on flux balance analysis (20). Neutrophils cluster C2 was selected as PMN-MDSCs and 3 clusters (neutrophils C3, neutrophils C4, and neutrophils C5) as neutrophils without immunosuppressive function, based on the MDSC signature score (19). Meta-reactions were filtered for core reactions, defined as reactions with Recon 2 confidence of either 0 or 4. Spearman’s correlations between MDSC signature score and each individual meta-reaction were computed using individual cells within PMN-MDSCs and neutrophils.

### CUT&Tag sequencing and data processing.

A total of 1 × 10^5^ PMN-MDSCs were collected after treatment with GC7 for 16 hours. Two replicates per group underwent CUT&Tag analysis using an antibody against H3K27ac. Genomic DNA extraction, library preparation, and sequencing were performed using the Hyperactive Universal CUT&Tag Assay Kit for Illumina Pro (Vazyme) according to the manufacturer’s protocol. In brief, PMN-MDSCs were resuspended in wash buffer and incubated with ConA Beads Pro (Vazyme) at room temperature for 10 minutes, then incubated with primary antibodies at 4°C overnight followed by secondary antibodies at room temperature for 1 hour. The samples were washed and incubated with pA/G-Tnp Pro at room temperature for 1 hour for fragmentation (Vazyme). Subsequently, DNA extraction was carried out using DNA extract Beads Pro, followed by PCR amplification with indexing primers (Vazyme). Purification of the PCR products was achieved using VAHTS DNA Clean Beads (Vazyme), and sequencing was conducted on the Illumina platform (NovaSeq 6000) at a sequencing depth of 12 gigabases per sample.

FastQC (v0.11.8) software was used for quality control (https://www.bioinformatics.babraham.ac.uk/projects/fastqc/). Then we mapped reads to mm10 mouse genome assembly with Bowtie 2 (v2.4.4) (http://bowtie-bio.sourceforge.net/bowtie2/index.shtml). SAMtools (v1.14) was used for quality filtering (http://www.htslib.org). After file format conversion, peak calling was performed by Macs2 (v2.2.7.1) with macs2 callpeak (https://github.com/taoliu/MACS). To identify regions that were differentially enriched between groups, we used DiffBind with a fold change cutoff of 0.5 and *P* value less than 0.05 (https://bioconductor.org/packages/release/bioc/html/DiffBind.html). Heatmaps were generated using DeepTools package (v3.5.1) (https://deeptools.readthedocs.io/en/latest/). ChIPseeker was used to perform annotation and functional enrichment analysis for differential genes between groups (https://www.bioconductor.org/packages/release/bioc/html/ChIPseeker.html). BigWig (BW) files were visualized using Integrative Genomics Viewer (v2.16.2) (https://igv.org/doc/desktop/).

### Statistics.

The results are presented as mean ± SEM. Comparisons between 2 groups were assessed using unpaired, 2-tailed Student’s *t* test, while comparisons among multiple groups were evaluated using 1-way ANOVA with Tukey’s multiple-comparison test. Grouped data with 2 independent categorical variables were analyzed using 2-way ANOVA with Šidák’s multiple-comparison test. Survival curves were generated and statistical analysis conducted using the Kaplan-Meier method and log-rank test. Correlation analysis was assessed using Spearman’s correlation coefficient. Statistical significance was determined using GraphPad Prism (v10.1.2) software. A *P* value less than 0.05 was considered significant.

### Study approval.

The human studies were approved by the Clinical Ethics Review Board of Children’s Hospital of Fudan University; Tianjin Children’s Hospital; and Guangzhou Women and Children’s Medical Center. All animal studies were approved by the Institutional Animal Care and Use Committee of Tianjin Medical University, and conducted in compliance with the Ethical guidelines including principles of animal welfare (freedom from thirst and hunger; freedom from discomfort; freedom from pain, injury, and disease; freedom to express most normal behaviour; freedom from fear and distress) and principles of the 3Rs (replacement, refinement and reduction).

### Data availability.

Values for all data points shown in graphs are reported in the Supporting Data Values file. The RNA sequencing data (GSE287178) and CUT&Tag data (GSE287179) were deposited in the Gene Expression Omnibus database.

## Author contributions

JZ conceived and supervised this study. JC performed the experiments, analyzed the data, and wrote the manuscript. L Zhu participated in most of the experiments. ZC, YZ, DZ, L Zhang, and PZ participated in animal model and flow cytometry analysis. RJ, BH, WZ, and JX recruited human subjects. WM, HW, ZY, QL, and YY provided suggestions in project design. JZ wrote the manuscript with input from all authors.

## Supplementary Material

Supplemental data

Supporting data values

## Version 1 04/01/2025

Electronic publication
