# Supplementary material for: Spermidine restricts neonatal inflammation via metabolic shaping of polymorphonuclear myeloid-derived suppressor cells
Source: J Clin Invest. 2025 Apr 1;135(7):e183559. doi: 10.1172/JCI183559 (PMC11957691; doi:10.1172/JCI183559)
Supplement: Supplemental data [file jci-135-183559-s081.pdf]

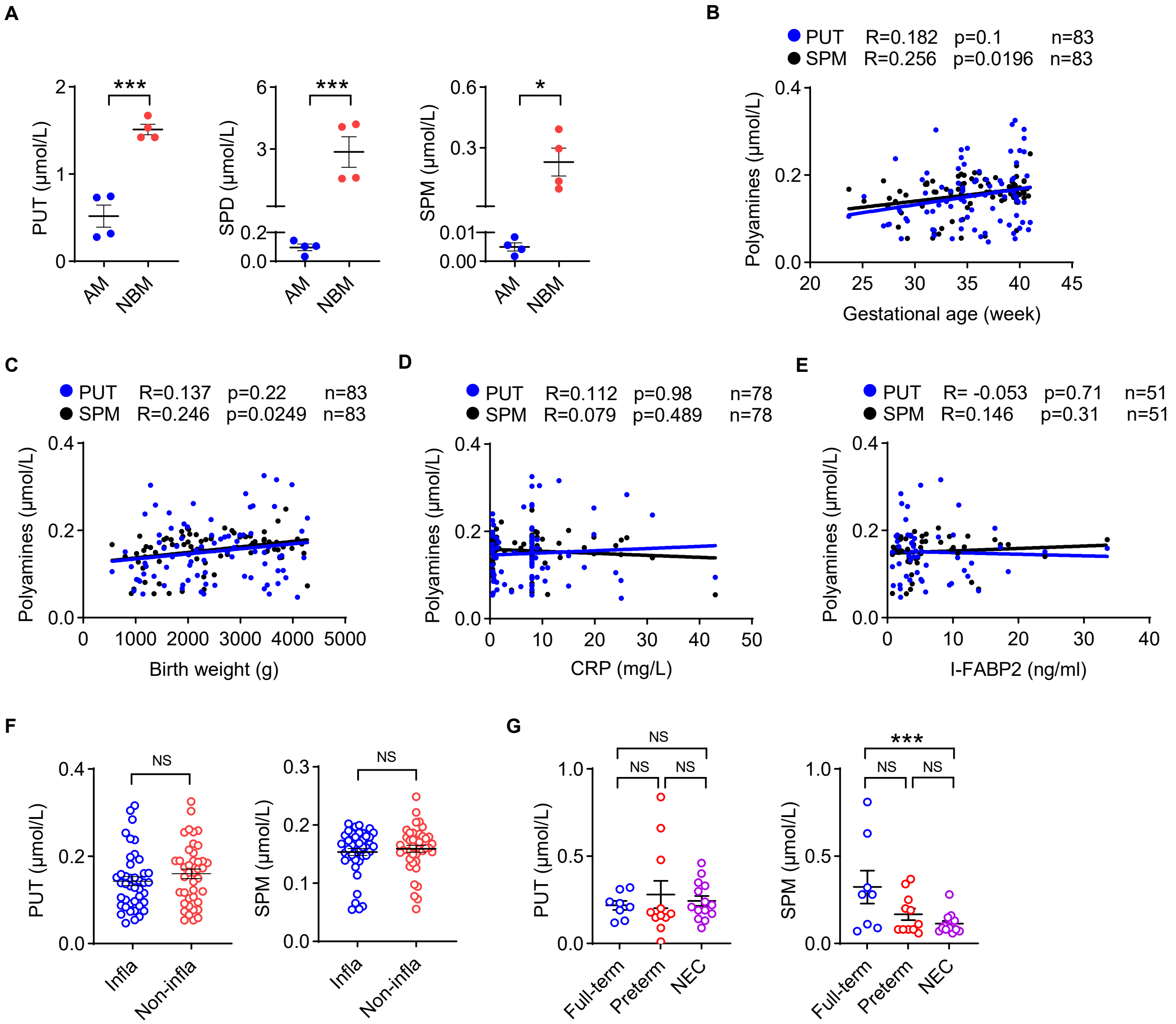

Supplemental Figure 1. Plasma spermidine is correlated with a reduced risk of inflammation in human newborns. (A) Plasma polyamines in adult mice (AM) and newborn mice (NBM) were detected by UPLC-MS/MS (n=4 per group). **(B-E)** Correlation between polyamines and gestational age (B), birth weight (C), C-Reactive Protein (CRP) (D), and I-FABP2 (E). Clinical parameters were listed in Supplemental Table 2. PUT, putrescine; SPM, spermine. **(F)** Plasma samples were collected from healthy neonates within 2 days of birth. The infants were divided into two groups based on the presence of subsequent inflammatory disorders within one month of follow-up: the inflammation group and non-inflammation group. PUT and SPM concentration in inflammation (Infla) (n=42) and non-inflammation (Non-infla) group (n=41). Clinical parameters were listed in Supplemental Table 2. **(G)** PUT and SPM concentration in healthy full-term babies (n=8), preterm babies (n= 11) and necrotizing enterocolitis (NEC) patients (n=14). Clinical parameters were listed in Supplemental Table 1. Data were shown as mean ± SEM. Statistical analysis was performed using unpaired two-tailed Student's t test (A and F), Spearman's correlation coefficient (B to E) and one-way ANOVA with Tukey's multiple comparison test (G). NS, not significant, $p > 0.05$; $*p < 0.05$, $**p < 0.01$, $***p < 0.001$.

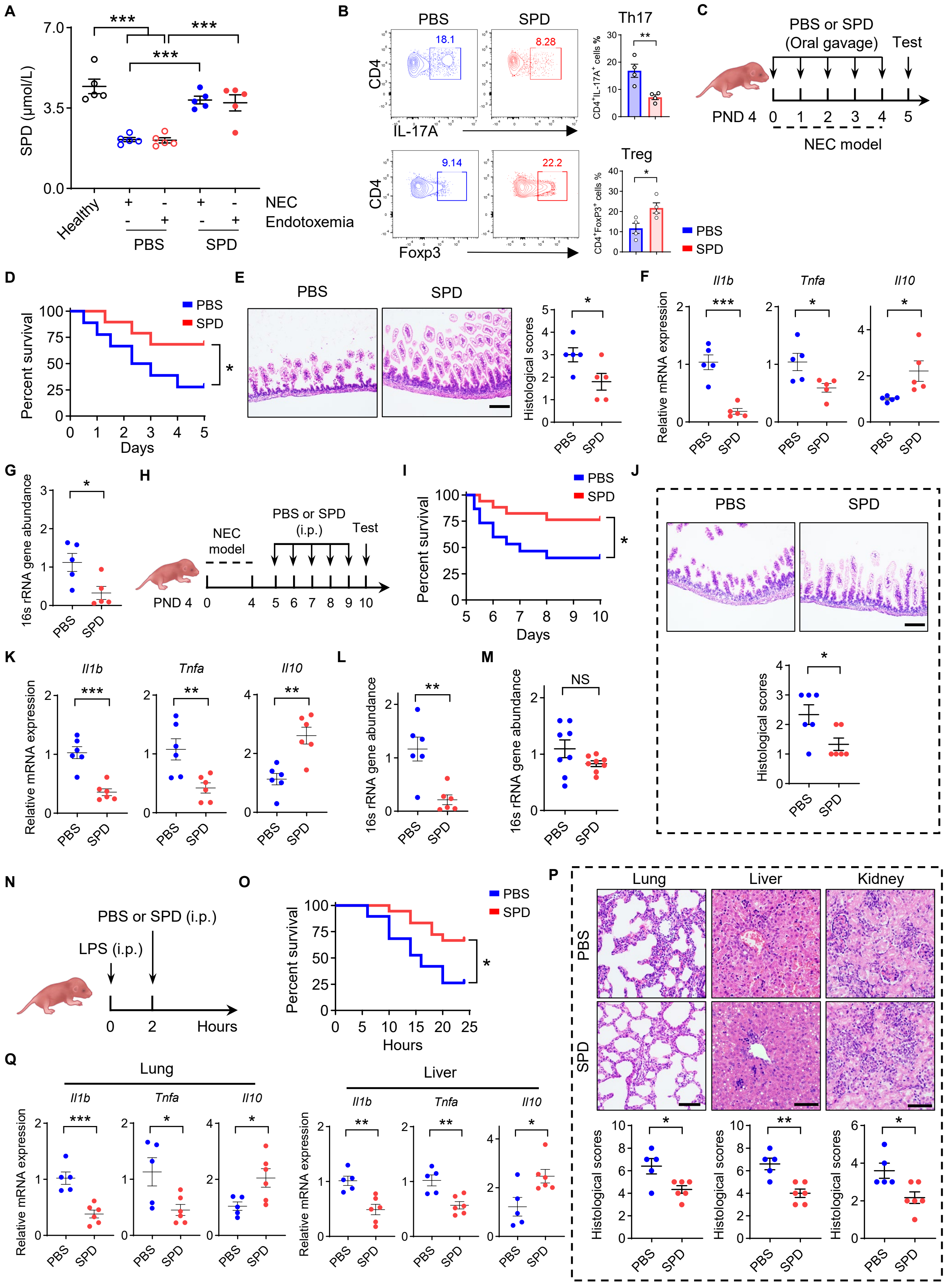
A
SPD (μmol/L)
7.0
3.5
0.0
***
***
***
Healthy
NEC
Endotoxemia
PBS
SPD
B
PBS
SPD
CD4
IL-17A
18.1
8.28
Th17
CD4+IL-17A+ cells %
**
Foxp3
9.14
22.2
Treg
CD4+FoxP3+ cells %
*
PBS
SPD
C
PBS or SPD
(Oral gavage)
Test
PND 4
0 1 2 3 4 5
NEC model
D
Percent survival
Days
PBS
SPD
*
E
PBS
SPD
Histological scores
*
F
Il1b
Tnfa
Il10
Relative mRNA expression
***
*
*
G
16s rRNA gene abundance
*
H
NEC
model
PBS or SPD
(i.p.)
Test
PND 4
0 4 5 6 7 8 9 10
I
Percent survival
Days
PBS
SPD
*
J
PBS
SPD
Histological scores
*
K
Il1b
Tnfa
Il10
Relative mRNA expression
***
**
**
L
16s rRNA gene abundance
**
M
16s rRNA gene abundance
NS
N
PBS or SPD (i.p.)
LPS (i.p.)
0
2
Hours
O
Percent survival
Hours
PBS
SPD
*
P
Lung
Liver
Kidney
PBS
SPD
Histological scores
*
**
*
Q
Lung
Liver
Il1b
Tnfa
Il10
Relative mRNA expression
***
*
*
**
**
*

Supplemental Figure 2. Spermidine attenuates neonatal inflammation. (A) Plasma sample were collected from healthy, NEC and endotoxemia mouse with or without SPD treatment. SPD was detected by ELISA (n=5 per group). **(B)** The proportions of Th17 and Treg in small intestine were detected by flow cytometry after NEC induction (n = 4 per group). Data are representative of 3 independent experiments. **(C-G)** Schematic approach for oral gavage of SPD (10 mg/kg/day) during induction of NEC (C) (PBS, n=18; SPD, n=19). The survival curve of mice (D). Data combined from 2 independent experiments. H&E staining and histopathological scores of intestinal tissues (E) (n = 5 per group). Relative mRNA expression of *Il1b*, *Tnfa* and *Il10* were determined by qRT-PCR (F) (n = 5 per group). Bacterial abundance in the intestinal wall was evaluated by 16s abundance (G) (n = 5 per group). **(H-L)** Schematic approach for treatment with SPD after induction of NEC (H) (PBS, n=15; SPD, n=17). The survival curve of mice (I). Data combined from 2 independent experiments. H&E staining and histopathological scores of intestinal tissues (J) (n = 6 per group). Relative mRNA expression of *Il1b*, *Tnfa* and *Il10* were determined by qRT-PCR (K) (n = 6 per group). Bacterial abundance in the intestinal wall was evaluated by 16s abundance (L) (n = 6 per group). **(M)** Healthy mice were treated with SPD (10 mg/kg/day, i.p.) for consecutive five days and bacterial abundance in intestine was evaluated by 16s abundance (n = 8 per group). **(N-Q)** Schematic approach for treatment with SPD after induction of neonatal endotoxemia (N) (PBS, n=19; SPD, n=18). The survival curve of mice (O). Data combined from 2 independent experiments. H&E staining and histopathological scores of lung, liver and kidney (P) (PBS, n=5; SPD, n=6). Relative mRNA expression of *Il1b*, *Tnfa* and *Il10* were determined by qRT-PCR (Q) (PBS, n=5; SPD, n=6). Data were shown as mean ± SEM. Statistical analysis was performed using unpaired two-tailed Student's t test (B, E-G, J-M, P and Q), two-way ANOVA with Sidak's multiple comparison test (A) and log-rank (Mantel-Cox) test (D, I and O). Scale bar indicates 100 μm. NS, not significant, $p > 0.05$; $^{*}p < 0.05$, $^{**}p < 0.01$, $^{***}p < 0.001$.

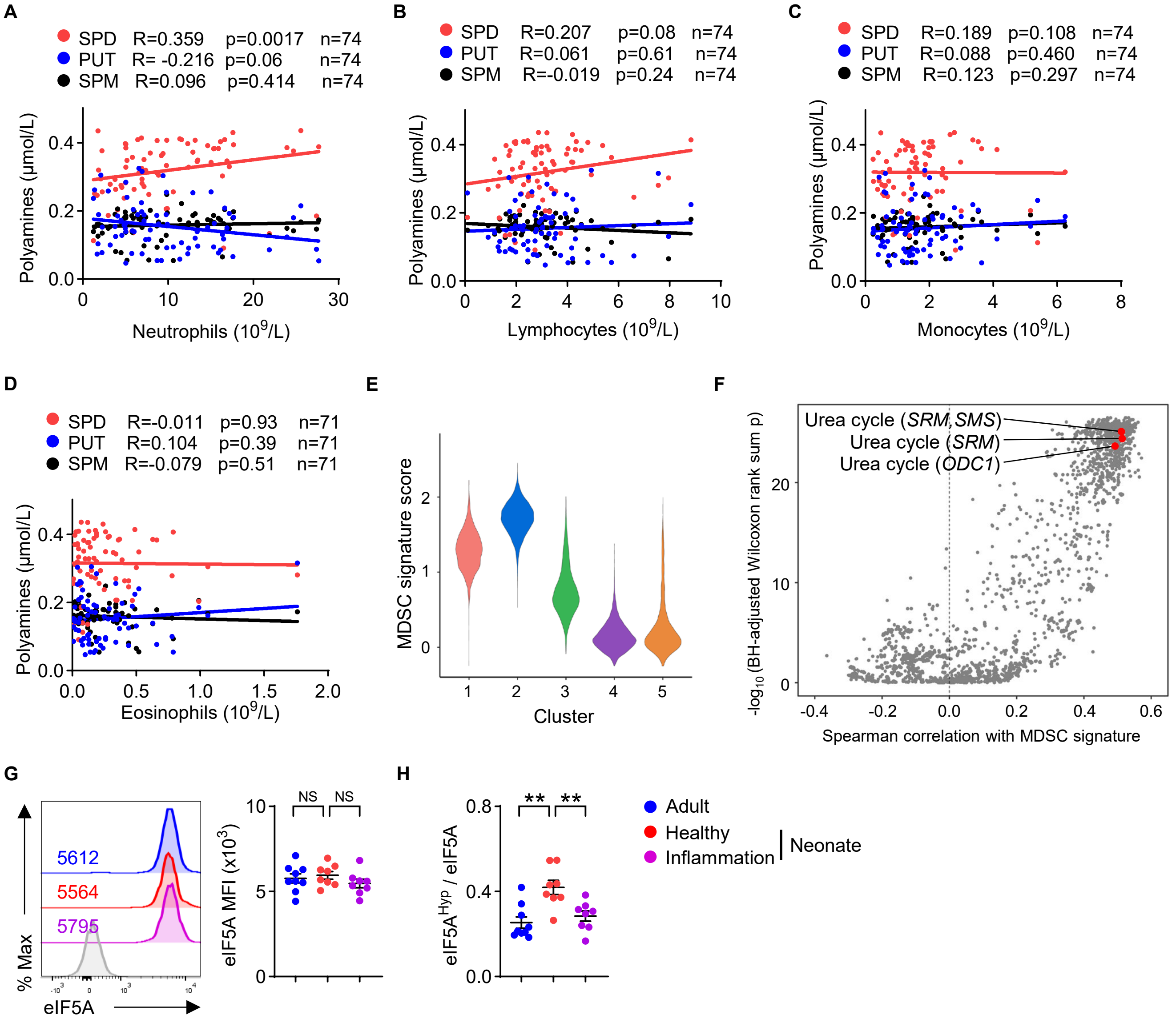

Supplemental Figure 3. Polyamine metabolism is enriched in neonatal myeloid cells. (A-D) Correlation between polyamines and absolute counts of neutrophils (A), lymphocytes (B), monocytes (C) and eosinophils (D). Clinical parameters were listed in Supplemental Table 2. **(E and F)** MDSC signature score of neutrophil subclusters in single-cell RNA sequencing dataset (GSE253963) (E). Correlation between polyamine metabolism reaction and MDSC signature (F). **(G and H)** The expression of eIF5A (G) and eIF5A^{Hyp}/eIF5A (H) in neutrophils from healthy adults, and PMN-MDSCs from healthy and inflammatory neonates were detected by flow cytometry. Clinical parameters were listed in Supplemental Table 1. Data were shown as mean ± SEM. Statistical analysis was performed using Spearman's correlation coefficient (A-D) and one-way ANOVA with Tukey's multiple comparison test (G and H). NS, not significant, $p > 0.05$; $*p < 0.05$, $**p < 0.01$, $***p < 0.001$.

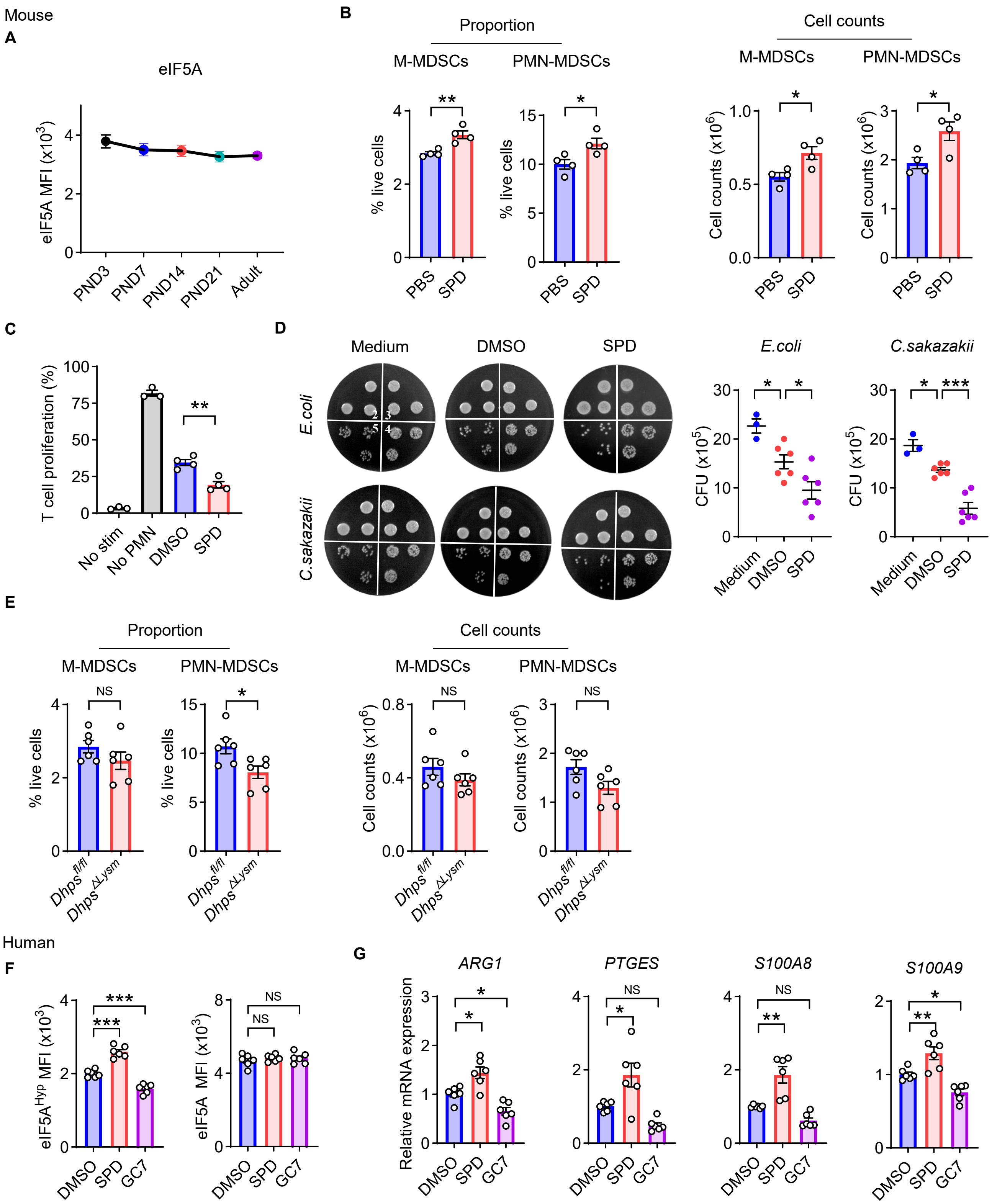

Mouse
A
eIF5A
eIF5A MFI (x10³)
PND3
PND7
PND14
PND21
Adult
B
Proportion
Cell counts
M-MDSCs
PMN-MDSCs
% live cells
Cell counts (x10⁶)
PBS
SPD
C
T cell proliferation (%)
No stim
No PMN
DMSO
SPD
D
Medium
DMSO
SPD
E.coli
C.sakazakii
CFU (x10⁵)
E
NS
Dhps fl/fl
Dhps ΔLysm
Human
F
eIF5A Hyp MFI (x10³)
eIF5A MFI (x10³)
GC7
G
ARG1
PTGES
S100A8
S100A9
Relative mRNA expression

Supplemental Figure 4. Polyamine-eIF5A^{Hyp} enhances the immunosuppressive activity of PMN-MDSCs in neonates. (A) The expression of eIF5A in CD11b^{+}Ly6C$^{lo/-}$Ly6G^{+} cells of different ages (n=4 per group). **(B** and **C)** 4-day-old mice were treated with SPD (10 mg/kg/day, i.p.) for five days. The proportion and absolute counts of splenic M-MDSCs and PMN-MDSCs were detected by flow cytometry (B) (n=4). Suppression of T cell proliferation by PMN-MDSCs (C) (n=4 per group). Data are representative of 3 independent experiments. **(D)** Spermidine-treated PMN-MDSCs and control PMN-MDSCs were incubated with *Escherichia coli* (*E. coli*) or *Cronobacter sakazakii* (*C. sakazakii*). Anti-bacterial activity was evaluated by calculating the colony-forming unit (CFU). Medium group was used as negative control, which was incubated without PMN-MDSCs. (Medium, n=3; DMSO, n=6; SPD, n=6). **(E)** The proportion and absolute counts of splenic M-MDSCs and PMN-MDSCs from *Dhps$^{fl/fl}$* and *Dhps$^{\triangle Lysm}$* littermates (n=6 per group). Data are representative of 3 independent experiments. **(F** and **G)** Human neonatal PMN-MDSCs were isolated from peripheral blood of healthy term babies. PMN-MDSCs were treated with SPD (0.2 μM) and GC7 (10 μM) for 16 hours. The expression of eIF5A^{Hyp} and eIF5A were detected by flow cytometry (F) (n=6 per group). Relative mRNA expression of genes involved in immunosuppressive function were detected using qRT-PCR (G) (n=6 per group). Clinical parameters were listed in Supplemental Table 1. Data were shown as mean ± SEM. Statistical analysis was performed using unpaired two-tailed Student's t test (B and E) and one-way ANOVA with Tukey's multiple comparison test (C, D, F and G). NS, not significant, $p > 0.05$; *$p < 0.05$, **$p < 0.01$, ***$p < 0.001$.

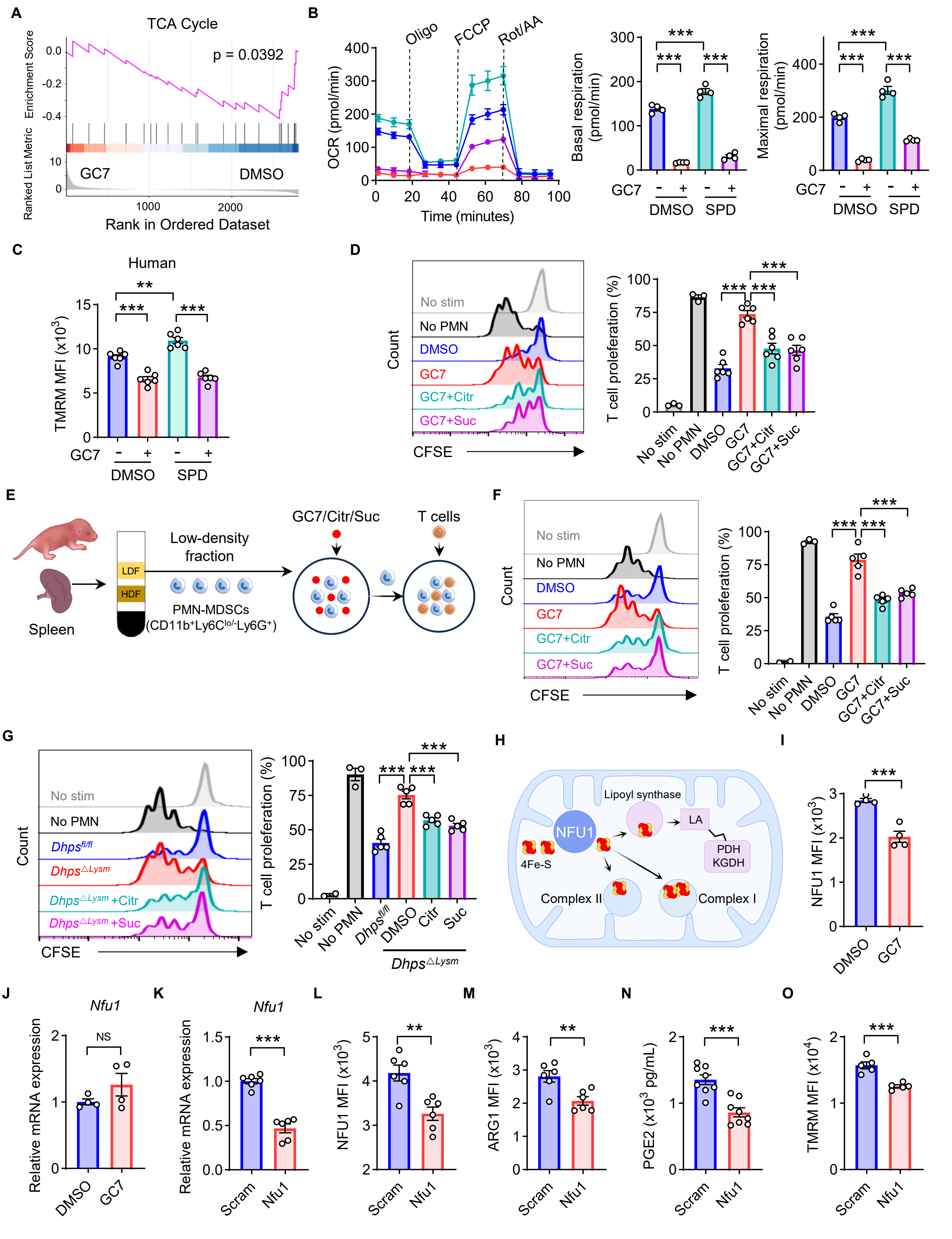

A
TCA Cycle
p = 0.0392
Enrichment Score
Ranked List Metric
GC7
DMSO
Rank in Ordered Dataset
B
Oligo
FCCP
Rot/AA
OCR (pmol/min)
Time (minutes)
Basal respiration (pmol/min)
Maximal respiration (pmol/min)
GC7
DMSO
SPD
C
Human
TMRM MFI (x10^3)
GC7
DMSO
SPD
D
No stim
No PMN
DMSO
GC7
GC7+Citr
GC7+Suc
Count
CFSE
T cell proleferation (%)
E
Spleen
LDF
HDF
Low-density fraction
PMN-MDSCs
(CD11b$^+$Ly6C$^{lo/-}$Ly6G$^+$)
GC7/Citr/Suc
T cells
F
No stim
No PMN
DMSO
GC7
GC7+Citr
GC7+Suc
Count
CFSE
T cell proleferation (%)
G
No stim
No PMN
Dhps$^{fl/fl}$
Dhps$^{\triangle Lysm}$
Dhps$^{\triangle Lysm}$ +Citr
Dhps$^{\triangle Lysm}$ +Suc
Count
CFSE
T cell proleferation (%)
DMSO
Citr
Suc
H
Lipoyl synthase
LA
NFU1
4Fe-S
PDH
KGDH
Complex II
Complex I
I
NFU1 MFI (x10^3)
DMSO
GC7
J
Nfu1
NS
Relative mRNA expression
DMSO
GC7
K
Nfu1
Relative mRNA expression
Scram
Nfu1
L
NFU1 MFI (x10^3)
Scram
Nfu1
M
ARG1 MFI (x10^3)
Scram
Nfu1
N
PGE2 (x10^3 pg/mL)
Scram
Nfu1
O
TMRM MFI (x10^4)
Scram
Nfu1

Supplemental Figure 5. eIF5A^{Hyp} maintains the mitochondrial function of neonatal PMN-MDSCs by modulating the translation of NFU1. (A) GSEA based on proteomics analysis. **(B)** Oxygen consumption rates (OCR) of PMN-MDSCs treated with SPD (0.2 μM), GC7 (10 μM) and SPD+GC7, was evaluated by seahorse assay (n=4 per group). **(C)** Human neonatal PMN-MDSCs were isolated from peripheral blood of healthy term babies. TMRM was detected after treatment with SPD (0.2 μM), GC7 (10 μM) for 16 hours (n=6 per group). Clinical parameters were listed in Supplemental Table 1. **(D)** Suppression of T cell proliferation by PMN-MDSCs, which were pretreated with GC7 (10 μM), GC7+citrate (Citr, 1 μM) and GC7+Succinate (Suc, 1 μM) (n=6 per group). Data are representative of 3 independent experiments. **(E-G)** Another strategy for isolating mouse PMN-MDSCs. The single-cell suspension of spleen was subjected to a discontinuous Histopaque (Sigma) gradient (1.077 and 1.119). Low-density fraction (LDF) was collected from the plasma-1.077 interface and PMN-MDSCs were isolated by FACS based on the gating of $CD11b^{+}Ly6C^{lo/-}Ly6G^{+}$ (E) . Suppression of T cell proliferation by PMN-MDSCs, which were pretreated with GC7 (10 μM), citrate (Citr, 1 μM) or succinate (Suc, 1 μM) (F). Data are representative of 3 independent experiments. Suppression of T cell proliferation by $Dhps^{fl/fl}$ and $Dhps^{\triangle Lysm}$ PMN-MDSCs, which were pretreated with citrate (Citr, 1 μM) or succinate (Suc, 1 μM) (G). Data are representative of 3 independent experiments. **(H)** NFU1 delivers [4Fe-4S] iron-sulfer cluster to target proteins and maintains their functions. (LA, lipoic acid; PDH, pyruvate dehydrogenase; KGDH, α-ketoglutarate dehydrogenase). **(I and J)** The expression of NFU1 in protein (I) and mRNA (J) level after treatment with GC7 (10 μM) (n=4 per group). Data are representative of 3 independent experiments. **(K-O)** Nfu1 was silenced by Nfu1-shRNA. The expression of Nfu1 was detected by qRT-PCR (K) and flow cytometry (L). ARG1 (M) (n=6 per group) and cellular PGE2 production (N) (n=8 per group) and TMRM (O) (n=5 per group) were detected. Data were shown as mean ± SEM. Statistical analysis was performed using one-way ANOVA with Tukey's multiple comparison test (D, F and G), and two-way ANOVA with Sidak's multiple comparison (B and C) and unpaired two-tailed Student's t test (I to O). NS, not significant; $p > 0.05$; $^{*}p < 0.05$, $^{**}p < 0.01$, $^{***}p < 0.001$.

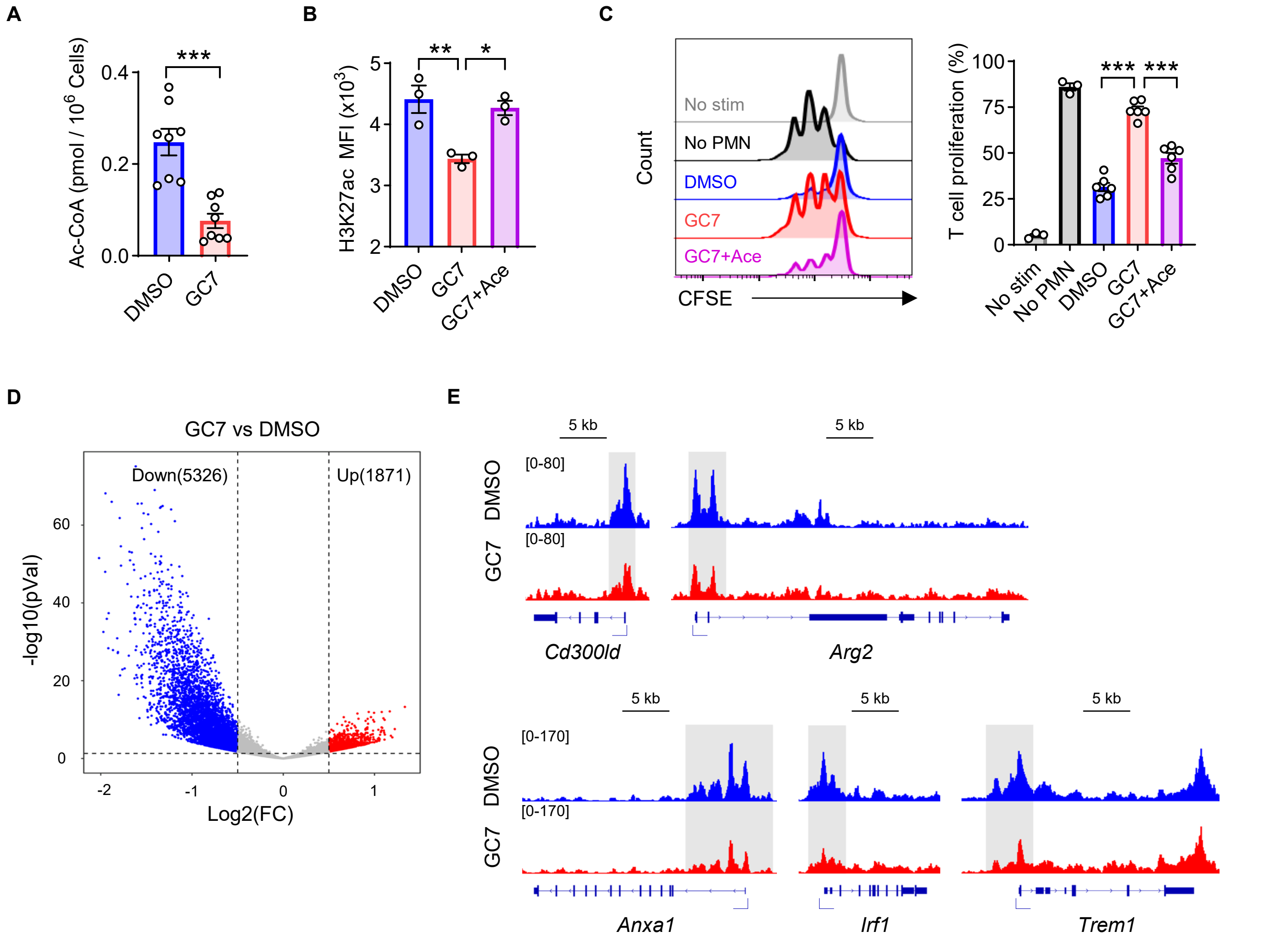

Supplemental Figure 6. Histone acetylation-mediated epigenetic regulation contributes to the effect of eIF5A^{Hyp} on PMN-MDSCs. (A) Cellular Ac-CoA in PMN-MDSCs were detected after treatment with GC7 (10 µM) for 16 hours (n=8 per group). **(B** and **C)** PMN-MDSCs were treated with GC7 (10 µM) and GC7+acetate (Ace, 1 µM). The levels of H3K27ac (B) (n=3 per group) and suppression of T cell proliferation (C) (n=6 per group) were detected after treatment. Data are representative of 3 independent experiments. **(D)** Volcano plot showing the differentially enriched peaks of H3K27ac. **(E)** Integrated Genomic Viewer screenshots for H3K27ac coverage at genes associated with immunosuppressive function of MDSCs. Statistical analysis was performed using unpaired two-tailed Student's t test (A) and one-way ANOVA with Tukey's multiple comparison test (B and C). NS, not significant, $p > 0.05$; $*p < 0.05$, $**p < 0.01$, $***p < 0.001$.

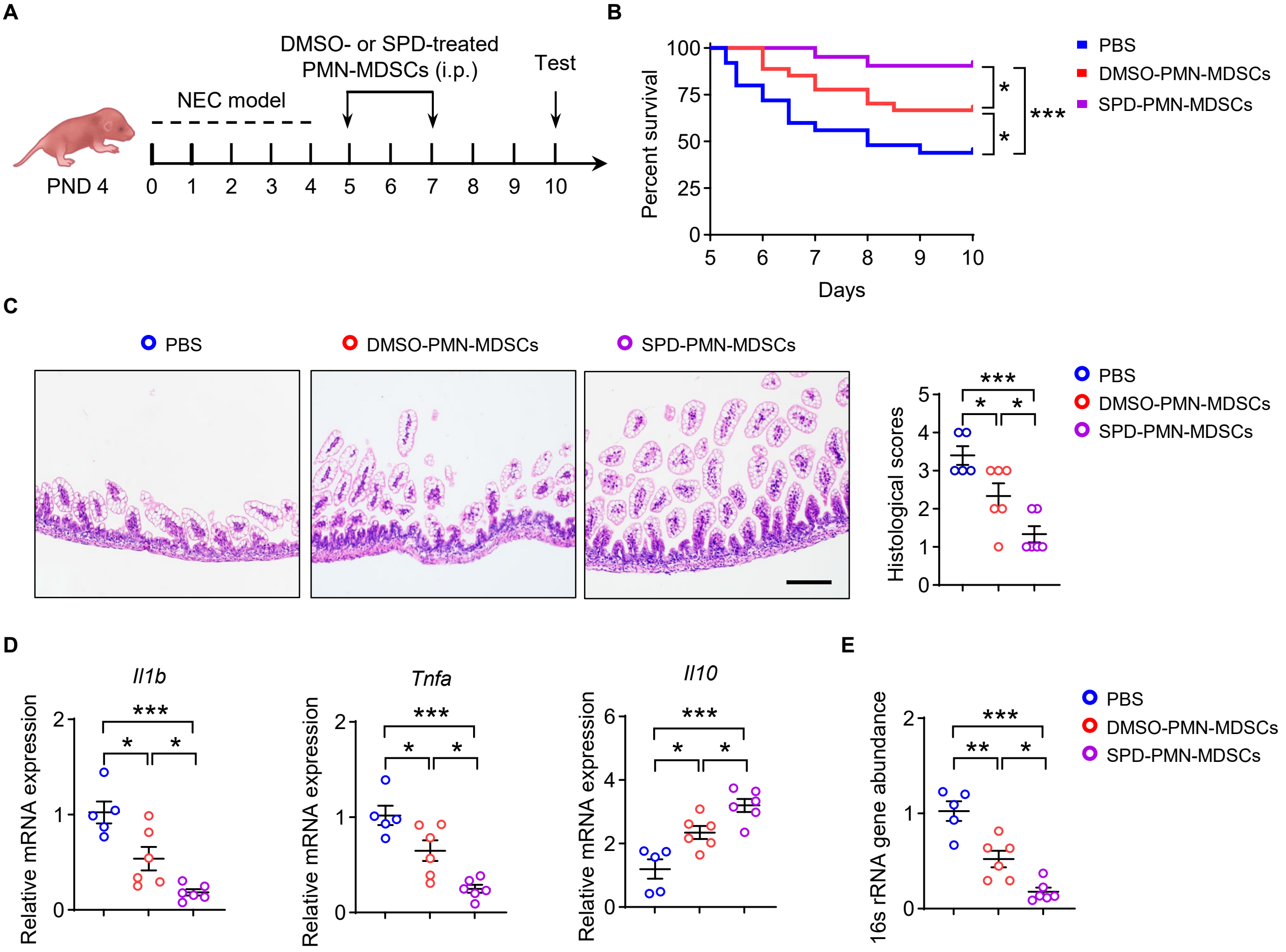

Supplemental Figure 7. Spermidine attenuates neonatal inflammation through eIF5A^{Hyp} in PMN-MDSCs. (A) Schematic approach for transfer of PMN-MDSCs after NEC induction. **(B)** The survival curve after induction of NEC (PBS, n=25; DMSO-PMN-MDSCs, n=27; SPD-PMN-MDSCs, n=21). Data combined from 2 independent experiments. **(C)** H&E staining and histopathological scores of intestine tissues. **(D)** Relative mRNA expression of *Il1b*, *Tnfa* and *Il10* were determined by qRT-PCR. **(E)** Bacterial abundance in the intestinal wall was evaluated by 16s abundance. Statistical analysis was performed using log-rank (Mantel-Cox) test (B) and one-way ANOVA with Tukey's multiple comparison test (C to E). Scale bar indicates 100 μm. NS, not significant, $p > 0.05$; $*p < 0.05$, $**p < 0.01$, $***p < 0.001$.

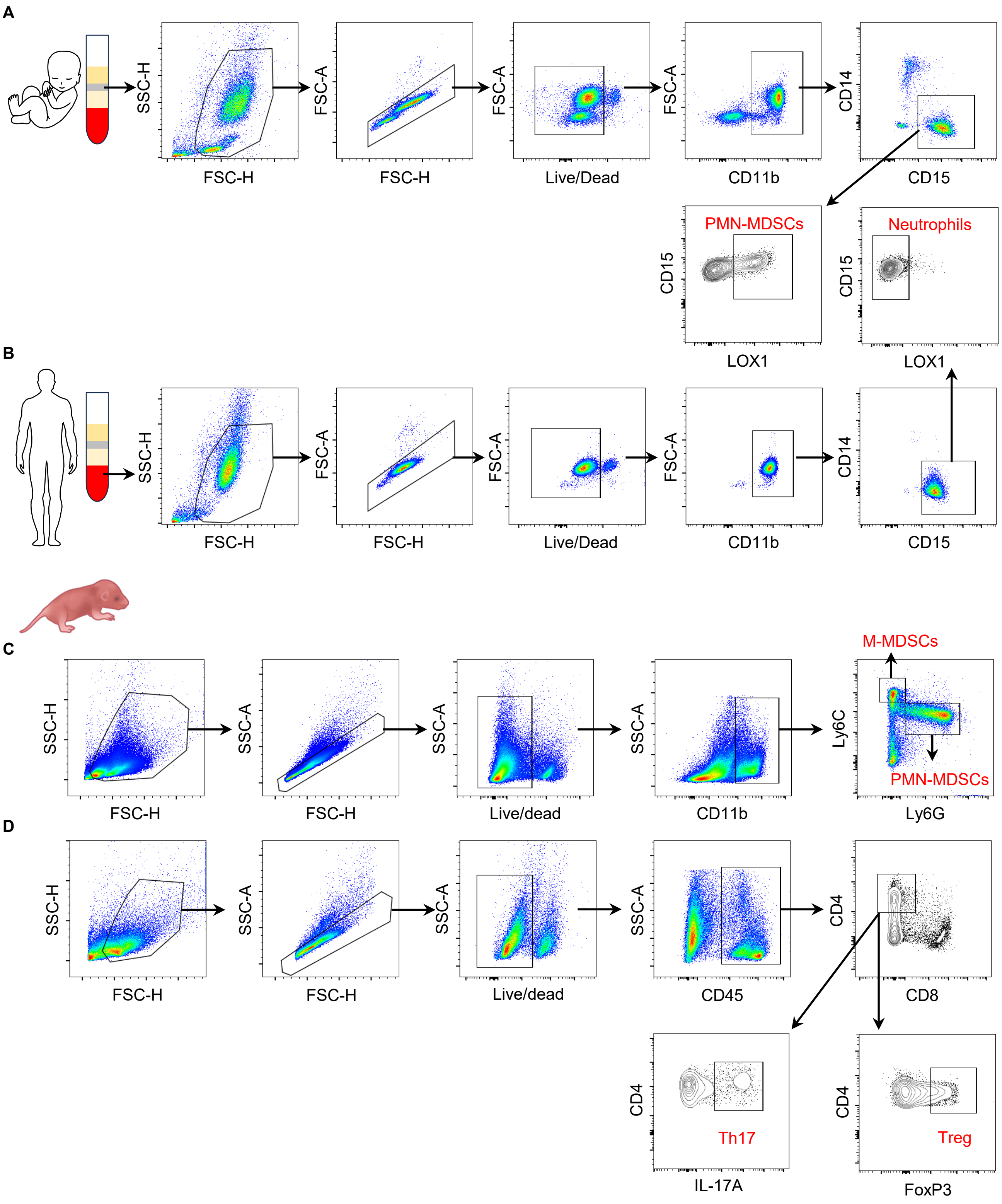

Supplemental Figure 8. Gating strategies. (A and B) Strategy for isolating neonatal PMN-MDSCs (CD11b$^+$CD14$^-$CD15$^+$LOX1$^+$) and adult neutrophils (CD11b$^+$CD14$^-$CD15$^+$LOX1$^-$) from human peripheral blood. Cell fractions were separated by Lymphoprep^{TM}. Mononuclear cell fraction (low density) (A) was collected for isolating PMN-MDSCs in neonatal sample and sediment (high density) (B) was collected for isolating neutrophils in adult sample. **(C)** Gating strategy for splenic immune cells of mice. **(D)** Gating strategy for intestinal immune cells of mice.

Supplemental Table 1. Clinical characteristics of Cohort 1.

Related to Figure 1A; Figure 1H; Figure 3, B-E; Supplemental Figure 1G, Supplemental Figure 3, G and H; Supplemental Figure 4, F and G; Supplemental Figure 5, C and D

Characteristic	Term	Preterm	Adult
**Demographics**	n=17	n=26	n=19
**Gender (M:F)**	8:9	12:14	9:10
**Gestational age, mean (SD), wk**	37.85±0.74	30.61±2.57	36.05±5.91 (Age: year)
**Birth weight, mean (SD), kg**	3.09±0.55	1.38±0.47	
**Cesarean (%)**	10 (58.82%)	18 (69.23%)	
**Indications for preterm delivery**			
**Maternal heart failure**		3 (11.54%)	
**Premature rupture of membranes**		6 (23.08%)	
**Preeclampsia**		7 (26.92%)	
**Fetal distress**		3 (11.54%)	
**Placental previa/abruption**		4 (15.38%)	
**Multiple pregnancies**		5 (19.23%)	
**Others**		3 (11.54 %)	
**Diseases**			
**Respiratory distress syndrome**	0 (0%)	10 (38.46%)	
**Sepsis**	1 (5.88%)	13 (50%)	
**Necrotizing enterocolitis**	4 (23.53%)	14 (53.85%)	
**Pneumonia**	1 (5.88%)	1 (3.85%)	

Supplemental Table 2. Clinical characteristics of Cohort 2.

Related to Figure 1, C-G; Supplemental Figure 1, B-E; Supplemental Figure 3, A-D

Characteristic	Inflammation group	Non-inflammation group
**Demographics**	n=42	n=41
**Gender (M:F)**	30:12	30:11
**Gestational age, mean (SD), wk**	34.33±5.03	36.38±2.96
**Birth weight, mean (SD), kg**	2.3±1.09	2.61±0.84
**Cesarean (%)**	26 (61.9%)	27 (65.85%)
**Diseases**		
**Respiratory distress syndrome**	22 (52.38%)	14 (34.15%)
**Sepsis**	20 (47.62%)	0 (0%)
**Necrotizing enterocolitis**	2 (4.76%)	0 (0%)
**Pneumonia**	8 (19.05%)	0 (0%)
**Urinary tract infection**	9 (21.43%)	0 (0%)
**Other inflammatory disorders**	10 (23.81%)	0 (0%)

Plasma samples were prospectively collected from healthy neonates within 2 days of birth. The infants were divided into two groups based on the presence of subsequent inflammatory disorders within one month of follow-up: the inflammation group and non-inflammation group.

Supplemental Table 3. Antibodies.

Antibodies	Source	Identifier
Anti-Mouse/human CD11b-FITC (clone M1/70)	BioLegend	Cat# 101206; RRID: AB_312789
Anti-Mouse/human CD11b-BV421 (clone M1/70)	BioLegend	Cat# 101235; RRID: AB_10897942
Anti-Mouse Ly6C-APC (clone HK1.4)	eBioscience	Cat# 17-5932-82; RRID: AB_1724153
Anti-Mouse Ly6C-PerCPCy5.5 (clone HK1.4)	eBioscience	Cat# 45-5932-82; RRID: AB_2723343
Anti-Mouse Ly6G-PE (clone 1A8-Ly6g)	eBioscience	Cat# 12-9668-82; RRID: AB_2572720
Anti-Mouse Ly6G-PE-Cy7 (clone 1A8)	Biolegend	Cat# 127618; RRID: AB_1877261
Anti-Mouse CD3-PE (clone 145-2C11)	eBioscience	Cat# 12-0031-85; RRID: AB_465498
Anti-Mouse CD45-APCCy7 (clone 30-F11)	BioLegend	Cat# 103116; RRID: AB_312981
Anti-Mouse CD4-biotin (GK1.5)	BioLegend	Cat# 100404; RRID: AB_312689
Anti-Mouse CD8-APCCy7 (clone 53-6.7)	BioLegend	Cat# 100714; RRID: AB_312753
Anti-Mouse CD8-PE (53-6.7)	BioLegend	Cat# 100708; RRID: 312747
Anti-Mouse-Hu/Mo Arginase 1-APC (clone A1exF5)	Invitrogen	Cat# 17-3697-82; RRID: AB_2734835
Anti-Human CD14-PE-Cy7 (clone 61D3)	Invitrogen	Cat# 25-0149-42; RRID: AB_1582276
Anti-Human CD15-eFluor™ 450 (clone HI98)	Invitrogen	Cat# 48-0159-41; RRID: AB_2016589
Anti-Human LOX-1-PE (15C4)	BioLegend	Cat# 358603; RRID: AB_2562181
Anti-Human LOX-1-APC (15C4)	BioLegend	Cat# 358605; RRID: AB_2563473
Anti-Mouse IL-17A-PE (TC11-18H10.1)	BioLegend	Cat# 506904; RRID_ 315464
Anti-Mouse FoxP3-APC (FJK-16s)	Invitrogen	Cat# 17-5773-82; RRID: AB_469457
Histone H3K27ac antibody mAb (clone D5E4)	Cell Signaling Technology	Cat# 8173S; RRID: AB_10949503
Rabbit anti-ODC1 Polyclonal antibody	Protein Tech	Cat# 28728-1-AP; RRID: AB_2881200
Rabbit anti-DHPS Polyclonal antibody	Protein Tech	Cat# 11184-1-AP; RRID: AB_2877751
Rabbit anti-NFU1 Polyclonal antibody	Protein Tech	Cat# 25598-1-AP; RRID: AB_2880151
Rabbit anti-EIF5A Polyclonal antibody	Abmart	Cat# TU256449S
Rabbit anti-Hypusine Monoclonal antibody (clone Hpu24)	AntibodySystem	Cat# RGK08101
Anti-Rabbit IgG H&L	Vazyme	Cat# Ab207-01
PE-Cy7 Streptavidin	Invitrogen	Cat# 25-4317-82
Donkey anti-rabbit Alexa Flour 647	Invitrogen	Cat# A-31573; RRID: AB_2536183

Supplemental Table 4. Reagents.

**Chemicals, peptides and recombinant proteins**	**Source**	**Identifier**
Alpha-difluoromethylornithine (DFMO)	MedChemExpress	Cat# HY-B0744D
GC7	MedChemExpress	Cat# HY-108314A
C646	MedChemExpress	Cat# HY-13823
5-Tetradecyloxy-2-furonic acid (TOFA)	MedChemExpress	Cat# HY-101068
Spermidine	Sigma-Aldrich	Cat# S2626
Acetate	Sigma-Aldrich	Cat# S8750
Sodium citrate	Sigma-Aldrich	Cat# 71498
Diethyl succinate	Sigma-Aldrich	Cat# 112402
Tetramethylrhodamine, methyl ester (TMRM)	Thermo Fisher Scientific	Cat# T668
MitoTracker™ Green FM	Invitrogen	Cat# M46750
Oligomycin	MedChemExpress	Cat# HY-N6782
Rotenone	MedChemExpress	Cat# HY-B1756
Antimycin A	MKBio	Cat# MS0070
Fluorocarbonyl cyanide phenylhydrazone (FCCP)	Sigma-Aldrich	Cat# C2920
Polybrene	YEASEN	Cat# 40804ES76
LIVE/DEAD Fixable Dead Cell Stain Kits	Thermo Fisher Scientific	Cat# L34966
4', 6-Diamidino-Phenylindole (DAPI)	Thermo Fisher Scientific	Cat# 62248
CellTrace™ CFSE	Invitrogen	Cat# C34554
Dithiothreitol (DTT)	Solarbio	Cat# D1070
Lipopolysaccharide (LPS)	Sigma-Aldrich	Cat# L2880
Lymphoprep™	Serumwerk Bernburg AG	Cat# 00319
SIINFEKL	Sigma-Aldrich	Cat# S7951
Hyperactive Universal CUT&Tag Assay Kit for Illumina Pro	Vazyme	Cat# TD904
TruePrep index Kit V2 for Illumina	Vazyme	Cat# TD202-00
Recombinant murine GM-CSF	Peprotech	Cat# 315-03-100
Recombinant human GM-CSF	Peprotech	Cat# 300-03
Mouse PGE2 ELISA kit	MEIMIAN	Cat# MM-0062M2
Seahorse XFe24 FluxPak	Agilent	Cat# 102342-100
Human putrescine ELISA kit	MEIMIAN	Cat# MM-92784201
Human spermidine ELISA kit	Mlbio	Cat# ml-E4461
Human spermine ELISA kit	Mlbio	Cat# ml-E5544
Mouse spermidine ELISA kit	Mlbio	Cat# YJ580071
Acetyl Coenzyme A ELISA kit	Elabscience	Cat# E-EL-0125
Human I-FABP2 ELISA kit	Boster	Cat# EK1410

Supplemental Table 5. RT-qPCR primers sequences.

Gene	Forward primer	Reverse primer	Species
S100a8	GGAAATCACCATGCCCTCT	TTTATCACCATCGCAAGGAAC	Mouse
S100a9	AATGGTGGAAGCACAGTTGG	GCTGATTGTCCTGGTTTGTG	Mouse
Ptges	GCACACTGCTGGTCATCAAG	ACGTTTCAGCGCATCCTC	Mouse
Arg1	ATTATCGGAGCGCCTTTCTC	ACAGACCGTGGGTTCTTCAC	Mouse
Nfu1	AGGCGGTTCTGTCATGTAGC	TTTGGGGTGTCTTGTGTTTGA	Mouse
Il1b	GCAACTGTTCCTGAACTCAACT	ATCTTTTGGGGTCCGTCAACT	Mouse
Il10	GCTCTTACTGACTGGCATGAG	CGCAGCTCTAGGAGCATGTG	Mouse
Tnfa	ATCGGTCCCCAAAGGGATGAGAAGTTC	GACGTGGGCTACAGGCTTGTCACTC	Mouse
16srRNA	GTGSTGCAYGYTGTCGTCA	ACGTCRTCCMCACCTTCCTC	Mouse
S100A8	ATGCCGTCTACAGGGATGAC	ACTGAGGACACTCGGTCTCTA	Human
S100A9	GGTCATAGAACACATCATGGAGG	GGCCTGGCTTATGGTGGTG	Human
PTGES	TCCTAACCCTTTTGTCGCCTG	CGCTTCCCAGAGGATCTGC	Human
ARG1	GTGGAAACTTGCATGGACAAC	AATCCTGGCACATCGGGAATC	Human
